# Sustainable Polymerization of Natural Lactones via Iron Catalysis: An Integrated Experimental and Computational Study

**DOI:** 10.1021/acssuschemeng.5c07947

**Published:** 2025-11-05

**Authors:** Giuseppe Gravina, Eugenio Romano, Alessia Liporace, Massimo Christian D’Alterio, Giovanni Talarico, Claudio Pellecchia

**Affiliations:** † Dipartimento di Chimica e Biologia “A. Zambelli”, 19028 Università Degli Studi di Salerno, Via Giovanni Paolo II 132, Fisciano, Salerno 84084, Italy; ‡ Dipartimento di Scienze Chimiche, 9307 Università Degli Studi di Napoli Federico II, Via Cintia 21, Napoli 80126, Italy; § Scuola Superiore Meridionale, Largo San Marcellino 10, Napoli 80138, Italy; ∥ Scuola Normale Superiore, Piazza dei Cavalieri 7, Pisa 56126, Italy

**Keywords:** ring opening polymerization of lactones, δ-substituted δ-lactones, ε-lactones, earth-abundant catalyst, DFT calculations of ROP

## Abstract

An earth-abundant Fe­(II)-based catalyst was employed for efficient and sustainable ring-opening polymerization of naturally occurring ε- and δ-lactones. High turnover frequencies (TOFs) and excellent control over the polymerization of δ-substituted δ-lactones demonstrate the catalyst’s ability to process monomers traditionally considered challenging or poorly polymerizable. Density Functional Theory (DFT) calculations suggest the formation of dinuclear iron species and underscore the mechanistic complexity of the system, including a monomer-dependent variation in the rate-determining step.

## Graphical abstract

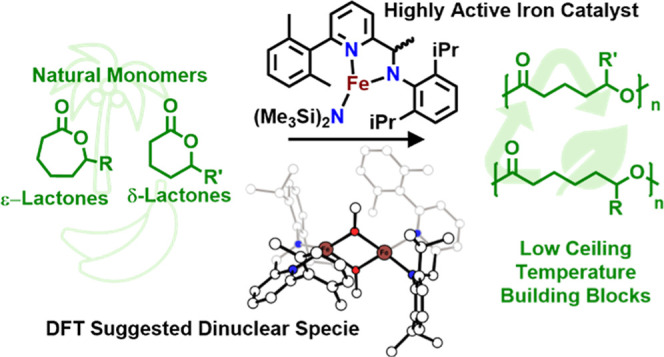

## Introduction

Plastics sustainability has become a prominent issue owing to the great impact of plastic waste mismanagement on environmental pollution.^1–4^ In particular, the persistence of conventional fossil-based polymers in the environment^5^ and their partial fragmentation have led to widespread microplastic contamination across ecosystems and even within the human body.^6^ This grim scenario is driving the transition toward a more sustainable polymer industry, aligned with the principles of a circular economy.^1^ Central to this shift is the development of viable alternatives to traditional plastics, including polymeric materials that not only derive from renewable resources but also exhibit improved end-of-life degradability, recyclability, or reusability. Biodegradable polymers have been already introduced in the market for nondurable or disposable applications.^4^ Among them, aliphatic polyesters have emerged as the most promising replacements for the commodity plastics. Polylactic acid (PLA) is by far the most important polymer in this class from the application standpoint,^7,8^ with a world installed production capacity close to 1 million tons/year. PLA is industrially produced by ring opening polymerization (ROP) of lactide, the dimeric lactone of lactic acid, which is derived from renewable resources, e.g., by fermentation of glucose, obtained in turn from starch from corn or sugar beet. However, some limitations in the PLA properties, such as its slow biodegradation in seawater and in soil under ambient conditions or the material’s brittleness, prompted investigating the polymerization and the copolymerization of other lactones. The goal is the development of materials with improved performance showing chemical recycling to monomer (CRM), an attractive alternative to transforming polymers back into monomers, and an ideal strategy for realizing a circular plastics economy.^9^ The polymerization thermodynamics greatly affect the practicability of the CRM approach: for ROP of lactones, the equilibrium monomer-polymer is related to the ring strain, and so polymerization is less favored for 5-membered and 6-membered rings. A successful ROP of “non-polymerizable” γ-butyrolactone operating at subambient temperature, to reduce the entropic penalty of the reaction, has been recently reported by Chen. By using reaction conditions such that the formed polymer precipitates, he shifted the equilibrium toward the polymer that then could be easily converted back to the monomer by heating at 220 °C.^10^ Several monomers based on γ-butyrolactone with a fused *trans*-cyclohexyl ring were later polymerized at room temperature, affording high-molecular-weight polymers that were recycled back to their monomer under mild conditions.^11^ Very recently, δ-caprolactone (δCL) polymers synthesized by organocatalysts were claimed as materials with an almost quantitative CRM as well as a component of a thermoplastic elastomer class with excellent mechanical properties based on PLLA-*b*-PδCL-*b*-PLLA.^12^ As a matter of fact, δ-substituted δ-lactones are also considered poorly polymerizable monomers, owing to both the low ring strain and to the substitution in the δ-position.^13^ Although their polymerization has been much less investigated with respect to ε-caprolactone (εCL) or β-butyrolactone, they have recently attracted increasing attention as natural products commercially available as fragrances in the cosmetic industry or as flavors in the food industry.^14^ Most of the reported ROP studies of δ-substituted δ-lactones used organocatalysts and low polymerization temperature to achieve significant conversion, while the literature examples of metal-catalyzed ROP are very limited.^13^ A few metal catalysts, such as lanthanum,^15^ neodimium,^16^ or strontium isopropoxide,^17^ have been reported to operate via a coordination–insertion mechanism, generally affording low number-average molecular weight (*M*_n_ < 6 kDa) and incomplete monomer conversions using rather long reaction times. Alkali metal alkoxides were also reported to afford low molecular weight polyesters of variously substituted δ-valerolactones,^18^ while the addition of urea cocatalysts allowed better ROP control, resulting in high molecular mass polyesters.^19^ In the framework of our research for new efficient catalysts based on nontoxic metals,^20–22^ very recently some of us reported that 3-coordinated pyridylamido Fe­(II) complexes are extremely active catalysts for the ROP of l-lactide and ε-caprolactone (εCL) maintaining a living polymerization character.^23^

In this study, we investigate the reactivity of pyridylamido Fe­(II) complexes in the ROP of less reactive lactone monomers, specifically δ-alkyl−δ-lactones, such as δ-hexalactone (δHL), δ-nonalactone (δNL), δ-undecalactone (δUDL) (Scheme 1a), and ε-decalactone (εDL, Scheme 1b) compared with εCL. Owing to its abundance, low cost, and nontoxic nature, iron represents an appealing alternative to traditional metal centers for the development of sustainable ROP catalysts. Although several Fe complexes have been explored for the polymerization of cyclic esters^24^ their use remains less developed compared to Zn-based analogues. The design of iron complexes has involved a wide range of ancillary ligands, particularly polydentate N- and/or O-donor ligands, leading to species with diverse geometries and coordination numbers and showing variable catalytic performance. Among the most notable findings, tetra-coordinated (hybrid-guanidine)­FeCl_2_ complexes have been reported as highly active catalysts for the bulk ROP of technical-grade *rac*-LA and recrystallized L-LA at 150 °C, achieving activities comparable to Sn­(Oct)_2_ under identical conditions.^25^ By contrast, penta- or hexa-coordinated iron complexes supported by tridentate or tetradentate ligands generally exhibited lower activities.^24^ Exceptions include penta-coordinated LFeN­(SiMe_3_)_2_ species (L = tripodal pyridyl-amino-phenolate ligands), which demonstrated efficient and stereoselective catalysis of *rac*-LA ROP at room temperature. Low-coordinate iron catalysts are relatively rare, and one example is the three-coordinate (β-diiminate)­Fe­(O-*tert*-Bu), which proved to be an active catalyst for the ROP of lactide and caprolactone at room temperature.^26^ More recently, heteroleptic (amidomethylpyridine)­Fe­(II) N­(SiMe_3_)_2_ complexes were synthesized; although the three-coordinate species could not be isolated, their tetra-coordinated pyridine adducts were crystallographically characterized and tested in the ROP of L-LA. These complexes exhibited good activities in toluene at 50 °C, surpassing those of the in situ generated pyridine-free species.^27^

**Scheme 1:**
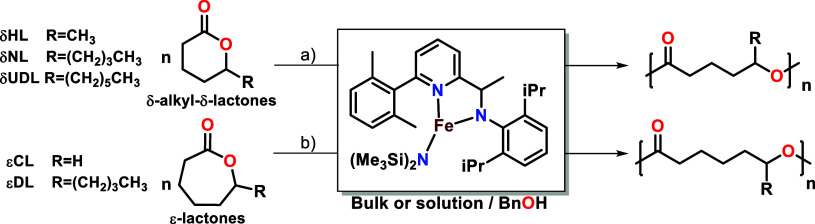
ROP of δ-Alkyl-δ-lactones (a) and ε-lactones (b)

Our goal is to identify catalysts that not only exhibit high activity but also enable controlled polymerization characterized by narrow dispersities (*D̵* = *M*_n_/*M*_w_ ≅ 1.1–1.2) being a key requirement for the synthesis of thermoplastic elastomer block copolymers with tunable mechanical properties. The presence of an alkyl side chain at the δ-position of these six-membered lactones significantly impacts both the thermodynamics and kinetics of polymerization. Compared to δ-valerolactone (*R* = H, Scheme 1), the ceiling temperature (*T*_c_) of δ-alkyl-substituted lactones drops markedly with increasing side chain length. Furthermore, polymerization becomes more kinetically challenging due to the formation of secondary alcohol during the propagation step, which hinders efficient chain growth.^28^

As we will demonstrate, the Fe­(II) catalysts investigated in this work exhibit the highest turnover frequencies (TOFs) reported to date for the ROP of δ-alkyl−δ-lactones (Scheme S1), while maintaining excellent control over polymerization with *D̵* consistently below 1.3 for the resulting homopolymers. To gain deeper mechanistic understanding, the experimental studies were complemented by detailed Density Functional Theory (DFT) analyses aimed at elucidating the influence of side-chain length on the polymerization pathway (Scheme 1). Several mechanistic scenarios were explored, explicitly considering the role of benzyl alcohol as an initiator, including the potential formation of dimeric catalyst–initiator adducts during the reaction.

## Experimental Section

### Experimental Details

All manipulations involving air- and moisture-sensitive compounds were performed under a nitrogen atmosphere in a Braun Labmaster glovebox or using Schlenk techniques. Glassware was dried in an oven at 120 °C overnight and exposed to vacuum–nitrogen cycles. All solvents were dried as follows: toluene was refluxed over metallic sodium and distilled under nitrogen before use. Deuterated solvents were purchased from Aldrich and stored in a glovebox over 4 A molecular sieves before use. The ε-decalactone, δ-hexalactone, δ-nonalactone, and δ-undecalactone were stirred with CaH_2_ for 24 h at room temperature and then distilled under reduced pressure before use. The pyridylamino ligand^29^ and the corresponding pyridylamido Fe­(II) complex^23^ were synthesized according to a previously published procedure. All other chemicals were commercially available and used as received, unless otherwise stated. NMR spectra were recorded using a Bruker Advance 400 or a 600 MHz Ascend 3 HD spectrometer. Chemical shifts (δ) are expressed as parts per million, and coupling constants (*J*) are in hertz. ^1^H NMR spectra are referenced using the residual solvent peak at δ = 7.26 for CDCl_3_, δ = 5.32 for CD_2_Cl_2_, and δ = 7.16 for C_6_D_6_. The molecular weights (*M*_n_ and *M*_w_) and the molecular weight distribution (*D̵*) of polymer samples were measured by gel permeation chromatography (GPC) using an Agilent 1260 Infinity Series GPC (ResiPore 3 μm, 300 × 7.5 mm, 1.0 mL min-1) and the refractive index (RI, PLGPC 220) detector. All measurements were performed with THF as the eluent at a flow rate of 1.0 mL/min at 35 °C. Monodisperse poly­(styrene) polymers were used as calibration standards. Differential scanning calorimetry (DSC) analysis was performed on a TA Q20. The instrument was calibrated for temperature and enthalpy by a high-purity indium (156.60 °C, 28.45 J × g^–1^) standard. The thermogravimetric (TGA) analysis was carried out on a Q500, from 25 to 800 °C at a heating rate of 20 °C, under a N_2_ flow.

### Ring Opening Polymerization (ROP) Procedure and Depolymerization Experiments

In a typical ROP run, a magnetically stirred reactor vessel was charged with a solution of the monomer in the selected solvent, to which the alcohol (benzyl alcohol) and then the Fe catalyst were added. Typically, 5 μmol of catalysts were used, and alcohol and monomer quantities described in the tables are referred to as the relative amount of that quantity. In bulk polymerization, the procedure was modified to eliminate the solvent. First, benzyl alcohol (BnOH) was dissolved in a volatile solvent, typically dichloromethane, and the required volume was then removed under a vacuum to isolate the desired amount. Next, the catalyst was weighed directly into the vial containing the BnOH, and finally, the monomer was added to initiate the reaction. After the prescribed time, an aliquot of the crude material was sampled and quenched in wet CH_2_Cl_2_ or CHCl_3_. The sample was subjected to monomer conversion determination, which was monitored by integration of monomer versus polymer in the ^1^H NMR spectrum (Figures S1, S3–S5). The reactions were quenched by exposure to air and adding wet CH_2_Cl_2_ or CHCl_3_. The crude products were precipitated in cold acidified methanol. The obtained polymers were collected by filtration and further dried in an oven at 60 °C under vacuum for 16 h. All polymers were characterized by ^1^H NMR and GPC analysis, and selected polymers also by DSC and TGA. Depolymerization experiments were performed by using 200 mg of polymer with 2 mol % of Sn­(Oct)_2_ (relative to the monomeric units) and glycerol ethoxylate as the alcohol, ensuring a [OH]/[cat.] ratio of 10:1. The experiment was carried out in a round-bottom flask connected to a cold trap immersed in liquid nitrogen under vacuum at 180 °C and quenched after 2 h by the addition of chloroform. Following solvent removal, an aliquot of the residue was dissolved in CDCl_3_ and analyzed by ^1^H NMR to determine its composition.

### Computational Details

Gaussian16 software^30^ was used for computational analysis. The geometry optimization and frequency analysis were conducted at the B3LYP^31,32^ level with the LANL2DZ^33^ basis set, including ECP for core electrons for Fe atoms and the SVP^34^ basis set for C, H, N, O, and Si. Single-point energy refinements are obtained, including solvent effects (PCM^35^ model, dichloromethane), dispersion effects (Grimme’s D3BJ)^36^ with SDD^37^ basis-set, including ECP for core electrons for Fe atoms and 6-311g­(d,p)^38,39^ for C, H, N, O, and Si. Benchmark calculations were carried out to validate the robustness of our strategy for the spin-state gaps and for critical points along the minimum energy path (MEP), using *meta*-GGA (M06L),^40^ hybrid GGAs (B3LYP-D3BJ, PBE0,^41,42^ and PBE0-D3BJ), hybrid *meta*-GGA (M062X,^40^ M06 ^40^ and TPSSh-D3BJ^43^), and hybrid range-separated (ωB97X-D).^44^ The DFT values are not corrected by translational entropies,^45^ and further details are reported in Supporting Information. The analysis of Non-Covalent Interactions (NCI) has been conducted through the software NCIPLOT 4.2 ^46^ and visualized through VMD.^47^

## Results and Discussion

### Polymerization of Poorly Reactive δ-Alkyl-δ-lactones

The heteroleptic [N,N^–^]­Fe^II^N­(SiMe_3_)_2_ complex was tested in the ROP of δHL, δNL, and δUDL, under a range of solvent-free conditions at 30 °C (Scheme 1a). The main polymerization conditions and results are summarized in Table 1.

**Table 1:** Experimental Results of δ-Alkyl-δ-lactones ROP Under Different Conditions

run^a^	mon	[M]/[Fe]/ [BnOH]	C (M)	*T* (°C)	*t* (h)	conv^b^ (%)	TOF (h^–1^)	*M*_nTheo_ ^c^ (kDa)	*M*_n,NMR_ ^d^ (kDa)	*M*_n,GPC_ ^e^ (kDa)	*D̵* ^e^
1	δHL	100:1:1	9.1	30	0.7	92	138	10.5		21.3	1.1
2	δNL	100:1:1	5.7	30	1	65	65	10.1	18.7	11.3	1.3
3	δUDL	100:1:1	5.2	30	1	50					
3					3	82	27	15.0	27.0	20.0	1.2
4	δHL	200:1:1	9.1	30	0.5	73	292	16.7	20.7	24.0	1.2
5	δHL	400:1:1	9.1	30	1.5	86	229	39.0		50.0	1.1
6	δNL	400:1:1	5.7	30	3	62	83	39.0	36.7	22.0	1.3
7	δUDL	400:1:1	5.2	30	3	41	42	39.0	39.0	30.0	1.2
					5	53					
8	δHL	800:1:1	9.1	30	4	81	162	77.0	66.2	58.7	1.1
9	δNL	800:1:1	5.7	30	18	70	31	87.0	44.4	26.7	1.2
10	δUDL	800:1:1	5.2	30	18	43	16	77.0	72.0	73.0	1.1
					26	52					
11^f^	δHL	100:1:1	2.0	30	0.5	64	128	8.1	6.6	15.7	1.4
12^f^	δNL	100:1:1	2.0	30	2	45	22	7.0	13.2	12.1	1.3
13^f^	δUDL	100:1:1	2.0	30	3	20	7	4.0	6.0	8.0	1.4
14^f^	δHL	400:1:1	4.0	30	3	80	73	37.0	31.0	26.7	1.1
15^f^	δNL	400:1:1	4.0	30	3	55	107	34.0	26.6	16.0	1.2
16^f^	δUDL	400:1:1	4.0	30	3	48	64	35.0	52.0	42.0	1.1
17	δHL	400:1:1	9.1	80	0.2	67	1600	31.0	26.6	11.7	1.6
18	δNL	400:1:1	5.7	80	0.5	54	432	34.0		22.4	1.3
19	δUDL	400:1:1	5.2	80	0.5	46	368	34.0	68.0	24.0	1.3

a Reactions were performed using 5 μmol of Fe catalyst and 1 equiv of BnOH.

b Monomer conversion was determined from ^1^H NMR spectra.

c Calculated *M*_n_ = Molecular mass of monomer × ([M]/[ROH]) × conversion.

d Calculated by the ratio of monomer/initiator values of integrations.

e Experimental *M*_n_ and *M*_w_/*M*_n_ (*D̵*) values were determined by GPC analysis in THF vs polystyrene standards.

f Toluene as the solvent.

In the first experiment (run 1, Table 1), δHL was polymerized using a δHL/Fe/alcohol molar ratio of 100:1:1, resulting in 92% monomer conversion after 40 min, approaching the expected equilibrium conversion under these conditions.^7^ Increasing the δHL/Fe ratio to 200 (run 4, Table 1) and a reaction time of 30 min led to a 73% conversion, with a 2-fold increase in turnover frequency (TOF). However, further increasing the ratio to 400:1 (run 5, Table 1) did not yield additional improvements in the TOF, although it remained high. Notably, the *M*_n_ of the resulting polymers increased proportionally with the monomer-to-catalyst ratio, rising from 21.3 to 24 and then to 50 kDa, while the *D̵* remained close to 1.0, indicating a well-controlled ROP process. In contrast, at an even higher δHL/Fe ratio of 800:1 (run 8, Table 1), the polymerization became less controlled, with 77% conversion after 4 h and a polymer with *M*_n_ = 58 kDa. A blank test using Fe­[(N­(SiMe_3_)_2_)]_2_ (the precursor used for the catalyst synthesis) resulted in very poor polymerization (18% conversion in 5 h at the plateau and bimodal molecular weight distribution).

A similar trend was observed in the polymerization of δNL and δUDL. Under solvent-free conditions, polymerization of δNL with 100 equiv of monomer (run 2, Table 1) resulted in 65% conversion within 30 min, with good control over molecular weight. Increasing the δNL/Fe molar ratio to 400:1 (run 6, Table 1) extended the reaction time to 3 h and led to 62% monomer conversion, still maintaining a well-controlled polymerization. However, further increasing the monomer-to-catalyst ratio (run 9, Table 1) required 18 h to achieve 70% conversion and yielded a polymer with a lower-than-expected molecular weight, suggesting a loss of control over the polymerization process. Despite this, the dispersity remained relatively low (*D̵* = 1.2), indicating that the polymer chains were still uniform in length.

For δUDL, polymerization at a monomer-to-catalyst ratio of 100:1 resulted in 50% conversion within 60 min, with excellent control over molecular weight (run 3, Table 1), indicating that the longer alkyl side chain does not significantly hinder the polymerization rate under these conditions. However, achieving conversions above 50% at higher monomer-to-catalyst ratios required extended reaction times: 5 h for δUDL/Fe = 400 (run 7, Table 1) and 26 h for δUDL/Fe = 800 (run 10, Table 1). Notably, the polymerizations remained well-controlled despite the prolonged reaction times, with *D̵* in the range of 1.1–1.2. To gain further insight into catalyst behavior in solution, additional polymerizations were conducted in toluene solution at 30 °C. At a fixed monomer concentration of 2 M and a monomer-to-catalyst ratio of 100:1, HL reached 64% conversion within 30 min (run 11, Table 1), while δNL required 2 h to achieve 45% conversion (run 12, Table 1), and δUDL reached only 20% in the same time frame (run 13, Table 1). These results highlight the decreasing reactivity trend with increasing side chain length under solution-phase conditions.

Polymerizations were next carried out at higher monomer concentrations ([monomer] = 4 M) with a monomer/catalyst ratio = 400:1. Under these conditions, a δHL conversion of 80% was obtained in 3 h (run 14, Table 1), still maintaining good control over molecular weight (*D̵* = 1.1).

In the same time frame, δNL and δUDL reached conversions of 55% (run 15, Table 1) and 48% (run 16, Table 1), respectively. When polymerizations were conducted under bulk conditions at 80 °C using the same 400:1 ratio, significantly faster reactions were observed: 67% of δHL (run 17, Table 1), 54% of δNL (run 18, Table 1), and 46% of δUDL (run 19, Table 1) were converted within just 10 min. However, these bulk polymerizations resulted in poorer control over molecular weights, likely due to increased viscosity and heat transfer limitations.

Polymerizations performed in a 2 M toluene solution at 80 °C did not proceed for any of the tested monomers, in line with thermodynamic expectations. The Gibbs energy of the ROP cycle is known to be strongly influenced by both the ring size and the nature and position of substituents,^14^ factors which reduce the driving force for polymerization under these dilute conditions.

The reported ROP of δ-alkyl-δ-lactones promoted by our iron catalysts achieved promising results: the TOFs achieved surpass those previously reported in the literature (Table S1), while the *D̵* remains consistently close to 1.0, indicating a well-controlled polymerization process suitable for the synthesis of well-defined polymer architectures. As a proof of concept, we synthesized di- and triblock copolymers of δHL with l-lactide (LA), combining soft blocks (PHL) with hard blocks (PLLA) (Table 2). Diblock copolymers were synthesized via a one-pot procedure, and δHL was sequentially added to a solution of benzyl alcohol. Once δHL polymerization was complete, a solution of LLA was introduced to form the second block (Scheme S1). The triblock copolymer using a similar procedure synthesized first the δHL middle block using 1,4-benzenedimethanol (BDM) as an initiator and then L-LA (Scheme S2).

**Table 2:** Synthesis of Di- and Triblock Copolymers

run^a^	sample	time (h)	*M*_n,Theo_ ^b^ (kDa)	*M*_n,GPC_ ^c^ (kDa)	*D̵* ^c^	*T*_g_ ^d^(°C)	*T*_m_ ^d^(°C)	Δ*H*_m_ ^d^(J/g)
1	PHL-*b*-PLLA [100-*b*-90]	5	21.0	35.5	1.2	–32	162	16
2	PLLA-*b*-PHL-*b*-PLLA [100-*b*-100-*b*-100]	3	36.8	65.7	1.3	–31	168	28

a Reactions were performed in 2 mL of CH_2_Cl_2_ using [Fe]_0_ = 5 mM and 1 equiv of BnOH (run 1) or BDM (run 2) at RT.

b Calculated *M*_n_ = Molecular mass of monomer × ([M]/[ROH]) × conversion.

c Experimental *M*_n_ and *M*_w_/*M*_n_ (*D̵*) values were determined by GPC analysis in THF using polystyrene standards.

d Determined by DSC in the first heating under a heating rate of 10 °C min^–1^.

The monomodal GPC elution profile and the narrow molecular-weight dispersity, together with the 2D DOSY NMR experiment (Figure S6), provided strong evidence for successful formation of the block copolymers. These results further support the living character of the ROP in this iron-catalyzed system.

### Polymerization of ε-decalactone

Encouraged by the promising results obtained in the ROP of δ-alkyl-δ-lactones, we extended our investigation to εDL (Scheme 1b), using the same Fe­(II) catalyst. εDL is a naturally occurring lactone featuring a seven-membered ring and an α-substituted side chain relative to the endocyclic ester, which is structurally analogous to δ-alkyl−δ-lactones. This substitution pattern introduces kinetic challenges during polymerization, as the resulting propagation step involves the formation of a secondary alcohol is inherently less favorable than primary alcohol formation and thus slows down the overall ROP process.

The results summarized in Table 3 indicate that the ROP of εDL proceeds slowly at 30 °C (runs 1–3, Table 3), requiring extended reaction times, particularly at higher monomer-to-catalyst ratios (e.g., 168 h in run 3, Table 3). However, the high thermal stability of our iron system enabled efficient polymerization at elevated temperatures. Indeed, at 80 °C, 70% monomer conversion was achieved within 1 h with good control over molecular weight (run 5, Table 3), while full conversion of 200 equiv was reached in just 15 min at 110 °C (run 6, Table 3), which turns into 45 min for a 79% conversion of 800 equiv of monomer (run 7, Table 3). High conversions with narrow dispersities were also obtained in toluene solution (runs 4 and 8, Table 3), confirming the robustness of the catalytic system under both bulk and solution-phase conditions. Compared to the unsubstituted ε-caprolactone, the TOFs achieved with εDL are moderate, reflecting the additional kinetic challenges imposed by the α-branching in the monomer structure.^13^

**Table 3:** Experimental Results for ROP of εDL Under Different Conditions

run^a^	mon	[M]/[Fe]/[ROH]	C (M)	*T* (°C)	*t* (h)	conv^b^ (%)	TOF (h^–1^)	*M*_n,Theo_ ^c^(kDa)	*M*_n,NMR_ ^d^ (kDa)	*M*_n,GPC_ ^e^ (kDa)	*D̵* ^e^
1	εDL	100:1:1	5.7	30	5	55	11	10	14	12	1.3
2	εDL	400:1:1	5.7	30	24	30	3	41	50	29	1.1
					48	44					
					72	61					
3	εDL	800:1:1	5.7	30	96	43	4	99	88	41	1.2
					120	58					
					168	73					
4^f^	εDL	400:1:1	4.0	30	24	78	8	65	61	44	1.1
					48	96					
5	εDL	400:1:1	5.7	80	1	70	280	48	52	36	1.0
6	εDL	200:1:1	5.7	110	0.25	100	800	34	38	29	1.2
7	εDL	800:1:1	5.7	110	0.75	79	843	108		47	1.1
8^f^	εDL	400:1:1	2.0	80	4.5	85	76	58	70	39	1.1

a Reactions were performed using 5 μmol of catalyst and 1 equiv of BnOH.

b Conversion of monomer was determined from ^1^H NMR spectra.

c Calculated *M*_n_ = Molecular mass of monomer × ([M]/[ROH]) × conversion.

d Calculated by the ratio of monomer/initiator values of integrations.

e Experimental *M*_n_ and *M*_w_/*M*_n_ (*D̵*) values were determined by GPC analysis in THF using polystyrene standards.

f Reactions performed in toluene solution.

The ^1^H NMR spectra of the polymers are collected in Figures S1–S5. The thermal characterization of the samples showing the *T*_g_ spanning in the range of −37 to −68 °C are reported in Table S3. The low *T*_g_ values confirm the use of such materials as soft segments for the design of thermoplastic elastomers. Finally, as established in the introduction, these lactones are suitable for CRM, an attractive alternative to transforming polymers into monomers. We selected a poly­(δHL) (entry 1, Table 1) and a poly­(εDL) (sample 7, Table 3) polymer for depolymerization experiments (further details are reported in Supporting Information) using 2 mol % of Sn­(Oct)_2_ as a catalyst in the presence of 10 equiv of glycerol ethoxylate. The reactions, conducted under vacuum at 180 °C, afforded a nearly quantitative depolymerization for δHL and 73% of εDL at the same time and conditions (Figures S7 and S8).

### Kinetic Studies of δ-Alkyl-δ-lactones and ε-decalactone Polymerizations

A detailed kinetic study of δHL polymerization was performed by NMR, collecting aliquots at increasing time intervals, to determine the kinetic order of the polymerization and the apparent propagation rate constant (*k*_app_). In the first experiment, 100 equiv of δHL were polymerized in toluene solution (2 M) using 1 equiv of BnOH as co-initiator. A plot of ln­([δHL]_0_/[δHL]_*t*_) versus time (Figure 1a) shows a first-order dependence on the concentration of δHL, with *k*_app_ = 1.00 ± 0.08 × 10^–3^ s^–1^ (*R* ^2^ = 0.98). The comparison between the molecular weight determined by NMR, based on the integration of the initiator and polymer signals over time, and the theoretical molecular weight calculated from monomer conversion is reported in Figure 1b. The close agreement between the two values confirms the good control achieved in the ROP, and the complete set of data is reported in Table S2.

**Figure 1:**
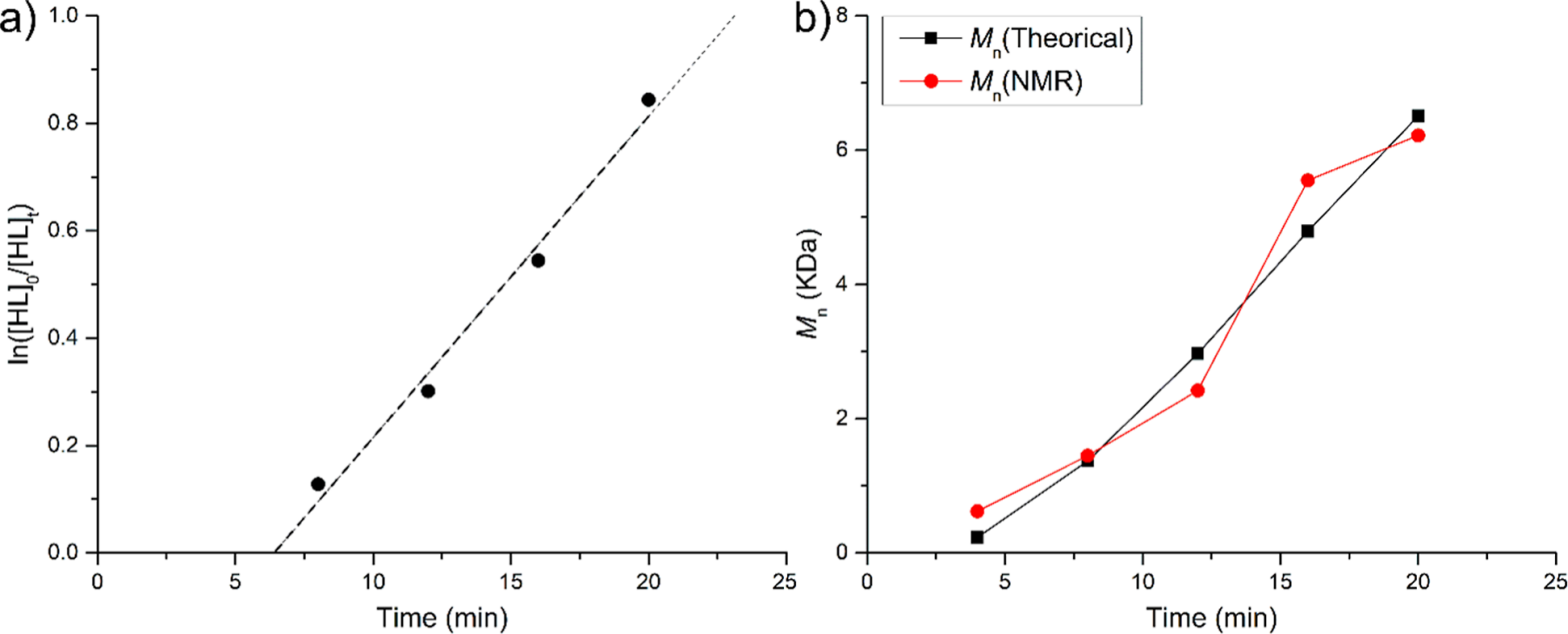
a) Pseudo-first-order kinetic plot for ROP of δHL promoted by system 1 ([Fe] = 5 mM; [δHL]/[Fe]/[BnOH] = 100:1:1; *T* = 30 °C; [δHL] = 2 M in toluene). (b) Comparison of *M*_n_ calculated by monomer conversion, and the experimental one calculated by ^13^C NMR spectra.

A detailed analysis of the kinetic profile in Figure 1a reveals a nonzero intercept, suggesting the presence of an induction period of approximately 6 min at the onset of polymerization. This unexpected behavior led us to hypothesize possible decomposition of the [N,N^–^]­Fe^II^(N­(SiMe_3_)_2_) precatalyst into its free proligand and other Fe­(II) species. To investigate this aspect, we conducted a series of polymerization experiments using Fe­(II) bis­(trimethylsilyl)­amide, the precursor employed in the catalyst synthesis, and/or the amine proligand in the presence of benzyl alcohol (see Supporting Information for details). While the combination of Fe­(II)­[N­(SiMe_3_)_2_]_2_ and one equivalent of BnOH resulted in some monomer conversion (19% after 24 h), the reaction afforded only oligomers with poor molecular weight control (*M*_n_ ≈ 0.6 kDa, *D̵* = 3.6). No polymerization was observed when only the proligand was used. These findings indicate that both BnOH and the [N,N^–^]­Fe­(II)­(N­(SiMe_3_)_2_) complex are essential for achieving efficient and controlled polymerization, supporting the formation of a catalytically active species in situ, most likely iron-alkoxide complexes.^48^

Interestingly, in all polymerization reactions, we observed the formation of a light blue precipitate at the early stages, which gradually dissolved as the reaction progressed. Given the known tendency of iron alkoxides to form multinuclear aggregates, we speculated that the formation of such species is responsible for the observed induction period.

To further probe this behavior, we conducted an experiment in which [N,N^–^]­Fe^II^(N­(SiMe_3_)_2_) was activated with benzyl alcohol to generate the characteristic blue species prior to monomer addition. While an induction period was still present, only 3% of monomer conversion was observed after 30 min, increasing to 72% after 2.5 h. However, the resulting polymer displayed a very low molecular weight (*M*_n_ ≈ 0.6 kDa) and a broad dispersity (*D̵* = 5.6), indicating poor control over the polymerization. These results suggest that, in the absence of monomer, multiple nonequivalent and possibly aggregated active species are formed, which are less effective in mediating controlled polymerization. In contrast, adding monomer during the formation of the active species seems to suppress the formation of such heterogeneous aggregates, thus favoring a more controlled polymerization process. Unfortunately, the paramagnetic nature of the Fe­(II) center precludes the use of in situ NMR spectroscopy to elucidate the active site structure during the reaction mechanism. To overcome this limitation, we turned to density functional theory (DFT) calculations to gain further insight into the nature of the active species and the mechanistic details of the ROP (see further section Mechanistic details by DFT calculations).

Due to the very low polymerization rates of δNL, δUDL, and εDL under the conditions previously employed for the kinetic study of δHL polymerization, a direct comparison of monomer reactivity required adjustment of the experimental setup. Reliable kinetic comparison across all monomers, including εCL as a reference, was achieved by using bulk polymerizations at 30 °C with a monomer/Fe/BnOH molar ratio of 100:1:1 (Figure 2). These conditions allowed for measurable reaction rates in all cases and thus enabled a consistent evaluation of monomer reactivities.

**Figure 2:**
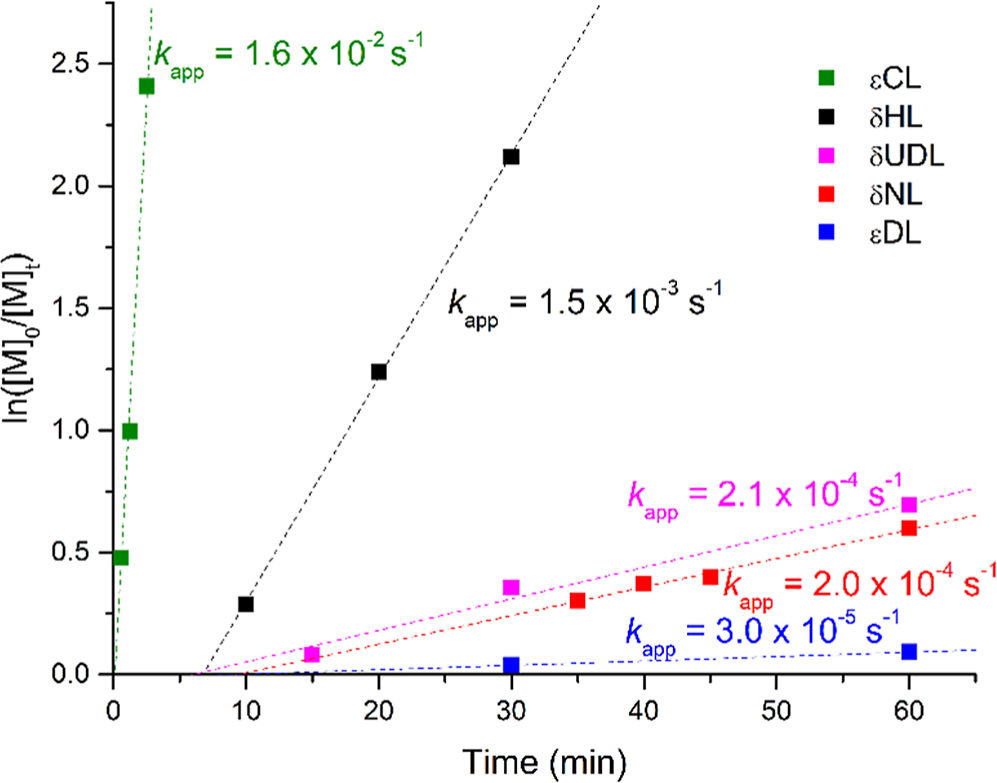
Pseudo-first-order kinetic plots for ROP of εCL (green, *R* ^2^ = 0.98), δHL (black, *R* ^2^ = 0.99), δUDL (pink, *R* ^2^ = 0.99); δNL (red, *R* ^2^ = 0.99), and εDL (blue, *R* ^2^ = 0.98) promoted by [M]/Fe/BnOH = 100:1:1; *T* = 30 °C.

Under these conditions, all polymerizations followed pseudo-first-order kinetics. The polymerization of δHL proceeded after an induction period of approximately 6 min, with an apparent rate constant *k*_app_ = 1.5 × 10^–3^ s^–1^. Both δNL and δUDL displayed similar rate constants (2 × 10^–4^ s^–1^ and 2.1 × 10^–4^ s^–1^), respectively, which are an order of magnitude lower than that of δHL. These monomers also exhibited induction periods of 9 min (δNL) and 6 min (δUDL). A comparable induction time has been calculated also for εDL, although the polymerization proceeded with a significantly lower rate constant (*k*_app_ = 3.0 × 10^–5^ s^–1^).

These results demonstrate how the length and position of the alkyl side chain in endocyclic esters play critical roles in determining polymerization kinetics. As expected, the unsubstituted εCL showed the highest reactivity, with a negligible induction period and a faster propagation rate, followed by δHL. In contrast, δNL and δUDL exhibited similar reactivity, suggesting that beyond a certain chain length, the steric influence of the side chain becomes less pronounced.^49^ Interestingly, despite having side chains of equal length (C4), εDL displays significantly lower reactivity compared to δNL. This difference is likely due to the distinct ring sizes (seven-membered in εDL and six-membered in δNL), suggesting that ring size and geometry significantly influence reactivity. We further sought to explain and rationalize these findings by using DFT calculations to decipher the rate-determining steps (RDS) involved in the ROP of δ-alkyl-δ-lactones and ε-lactones.

### Mechanistic Details by DFT Calculations

Two key observations prompted our interest and led us to undertake an in-depth mechanistic investigation based on DFT calculations for εCL, δHL, and δNL homopolymerizations. First, we aimed to understand why εCL uniquely lacks an induction period, as reported in Figure 2. Second, we sought to elucidate how variations in the side chain length at the δ- or ε-position influence the rate constants. We used the achiral model of the Fe­(II) catalyst reported in Scheme 1 by substituting the methyl group on the C bridging the pyridino and the amido moieties with a H atom to reduce the computational complexity deriving from the chirality. Nevertheless, due to the asymmetry of the ligand, the active species shows two different coordination sites (Figure 3). We used a molecular descriptor (%*V*_Bur_)^50,51^ to quantify the steric encumbrance around the metal center of the neutral precursor. Further details for the combined application of %*V*_Bur_ and DFT calculations applied in olefin polymerization catalysis^52,53^ and recently extended to ROP,^54,55^ are reported in Supporting Information. The analysis revealed a difference in steric map environment between the two coordination sites (called S_1_ and S_2_ for simplicity, Figure 3), revealing the higher steric congestion on the southern side, corresponding to the S_2_. This steric asymmetry likely directs the monomer (in the initiation step) and the growing chain (in the propagation step) toward the less hindered site, namely the NW quadrant. In particular, the NW quadrant is crucial for accommodating the monomer within the catalytic pocket.^56,57^ Figure S9 reports the steric maps of TS1 (nucleophilic attack) at both sites for εCL and δHL. The results indicate that the energy differences between the two sites are mainly related to the approach direction: both monomers preferentially approach from the NW side, thus avoiding the sterically hindered red zone in the SW quadrant. This effect is more pronounced for δHL than for εCL, due to the presence of the methyl substituent.

**Figure 3:**
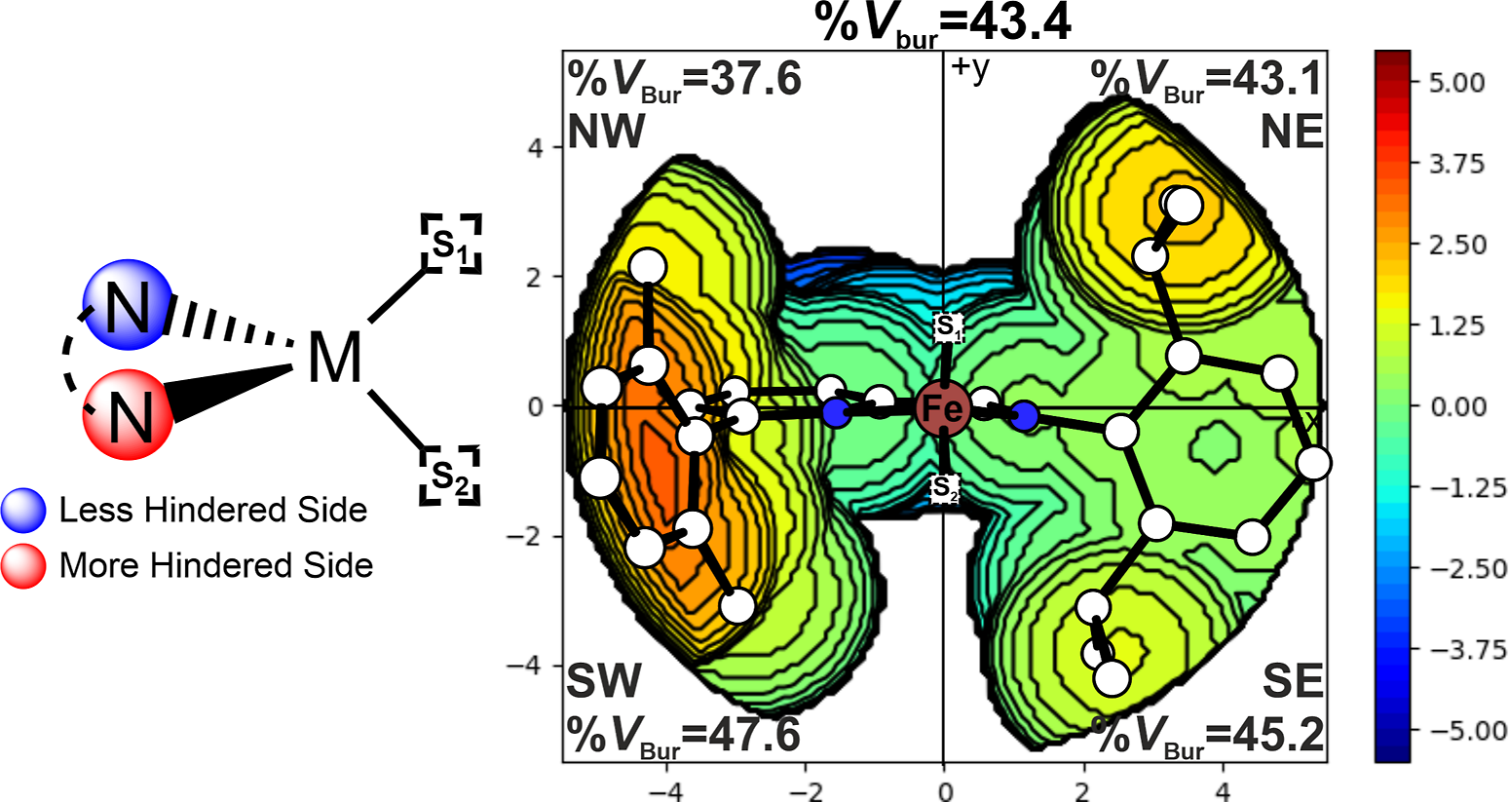
%*V*_Bur_ steric map of the neutral precursor with the two available coordination sites.

Notwithstanding the presence of two diastereotopic coordination sites, the intricacy of Fe­(II) complexes derives from the access to multiple spin states based on low-spin (singlet or triplet) or high-spin (quintet) states, whose potential energy surfaces (PESs) may be nearly degenerate. This requires, from a theoretical point of view, geometry optimizations for all accessible spin states and a comparison of their free energies to determine the most stable configuration. For the sake of readability, we anticipate here that the quintet spin state was found to be the most stable, and details for the energetics of the various spin states depending on the functionals used are reported in Table S4. Additionally, the stereochemistry of the monomers has been considered: indeed, the δHL is available as *R* and *S*, and each enantiomer can coordinate through two enantiofaces (si or re). The elements of chirality are summarized in Scheme S3, and in the following we report the results with the *S* enantiomers of δHL and δNL, and energetic details about the preferred enantioface are discussed in Table S5. The computational analysis for the ROP of εCL, δHL, and δNL has been conducted in both initiation and propagation steps by hypothesizing different polymerization mechanisms summarized in Figure 4.

**Figure 4:**
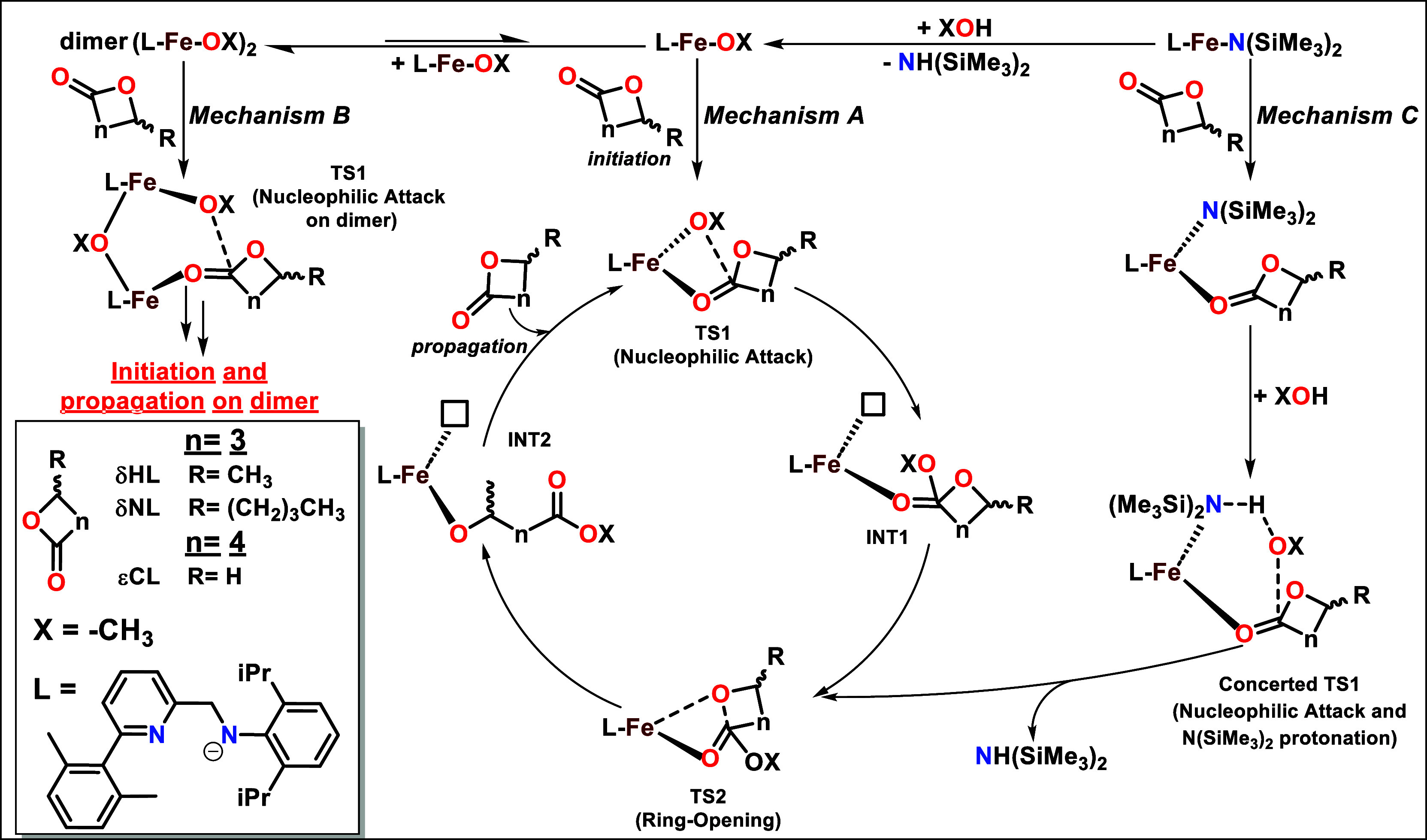
Mechanisms A, B, and C for ROP of εCL, δHL, and δNL computed at the DFT level in this study.

Let us discuss first the εCL polymerization following the Mechanism A hypothesis, whose Gibbs energies are reported in Figure 5, blue path. The neutral precursor reacts with one equivalent of alcohol (we used methanol (MeOH) in our calculations) to form a metal alkoxide intermediate (L–Fe–OMe) (Δ*G* = −7.1 kcal/mol). The Int1 then undergoes the initiation step, which involves two transition states (TSs): first, the nucleophilic attack of the alkoxide on the coordinated monomer (TS1), followed by ring-opening of the monomer (TS2), with the formation of a growing polymer chain bearing an alkoxide terminus. Int2 starts the propagation cycle having TS1′ as RDS (14.8 kcal/mol). Mechanism A is the “classical” coordination–insertion path under the assumption of a monomeric active species. However, in our opinion, this mechanism presents some weaknesses here summarized: (a) the presence of a precipitate at the early stages of polymerization processes gradually dissolving related to the observed induction periods (see previous section); (b) the formation of iron alkoxides in the form of multinuclear aggregates as recently reported in literature.^48^ Indeed, when we calculated the stability of the iron-alkoxide species, we found that the formation of homoleptic and dimeric complexes (Figure S10) is highly favored (Figure 5, black path). Their energetic stability and stoichiometry have been computed following two reaction schemes reported in the Supporting Information. Indeed, based on experimental evidence, the addition of alcohol to the neutral precursor solution leads to the formation of precipitates (with unclear composition).

**Figure 5:**
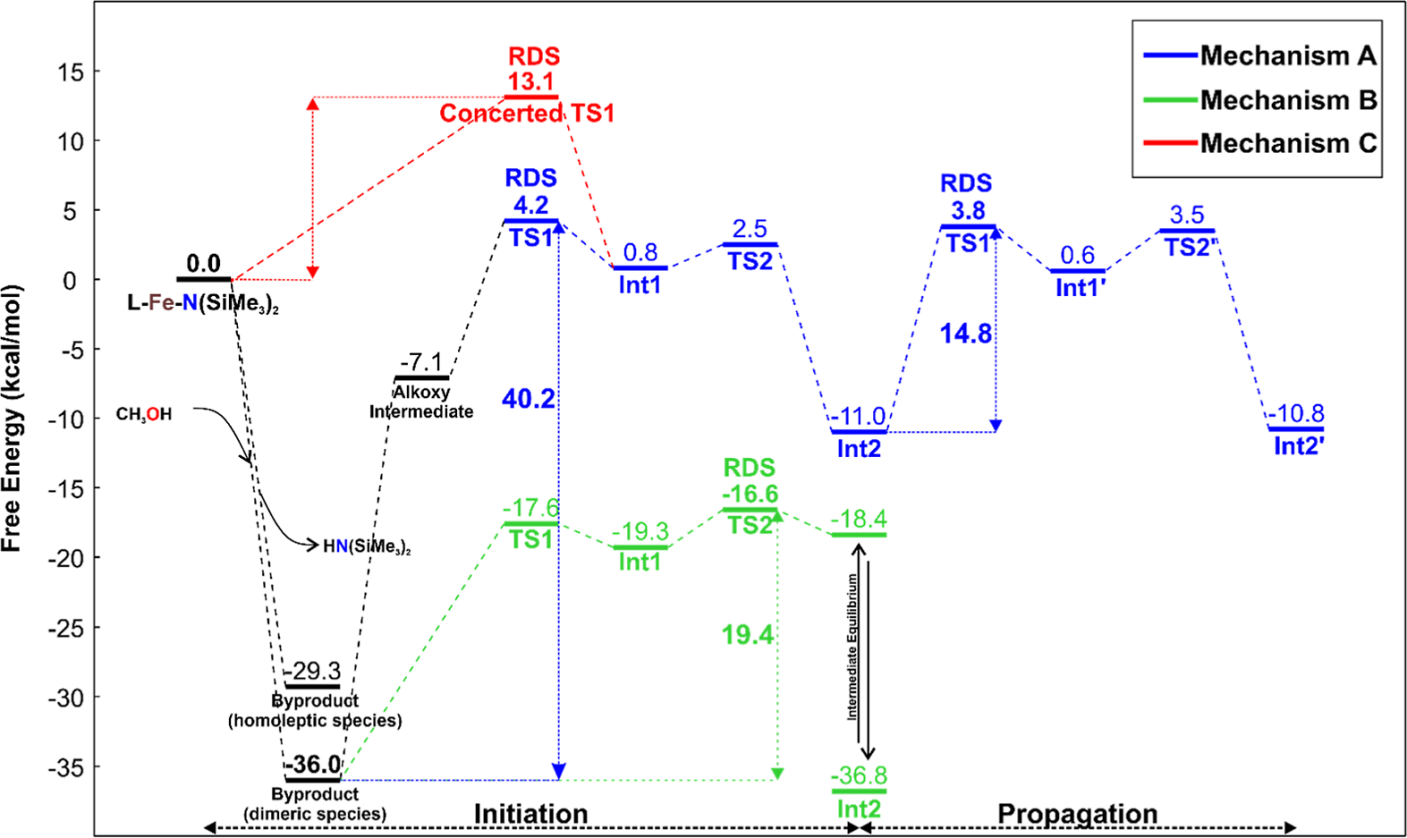
DFT-computed Gibbs energetic profiles for ROP of εCL (initiation and propagation steps) following Mechanism A (blue), B (green), and C (red). For discussion on the mechanisms see text.

We then calculated the Mechanism B sketched in Figure 4 under the assumption that, once the metal alkoxide species is formed, it can further lead to the generation of polynuclear adducts of the type (MOX)_*n*_. The DFT results are reported in Figure 5, green path, and for simplicity, only the dimeric species (MOX)_2_ are included in our computational model; however, the formation of higher-order species (*n* > 2) cannot be excluded. The activation barrier for the εCL initiation reaction is 19.4 kcal/mol, largely depending on the high stability computed for the dimeric species. The analogous MEPs for δHL and δNL are reported in Figure S11 and Table S6. The initiation barriers calculated for δHL, and δNL (22.6 and 21.7 kcal/mol, respectively) are higher than εCL and this result is interesting for two reasons: it accounts for the possibility that dimeric species themselves can initiate polymerization; the higher barrier of substituted species with bulky side groups to insert into dimeric adduct may support the presence of an induction period. The optimized DFT structures for TS1 and TS2 of εCL, δHL and δNL are reported in Figure 6, where the steric close contacts observed for δHL and δNL are drawn in red. It is worth noting that the RDS are changing, moving from εCL (TS2) to δHL and δNL (TS1), because the bulkiness of side chains exerts the main effect on the nucleophilic addition TS1 (Figure 6). Finally, the ring-opening event yields an open-chain intermediate, which can subsequently rearrange to form a highly stable O–Fe–O–Fe four-center dimer. This rearrangement to the more thermodynamically favorable structure is illustrated in Figure S12.

**Figure 6:**
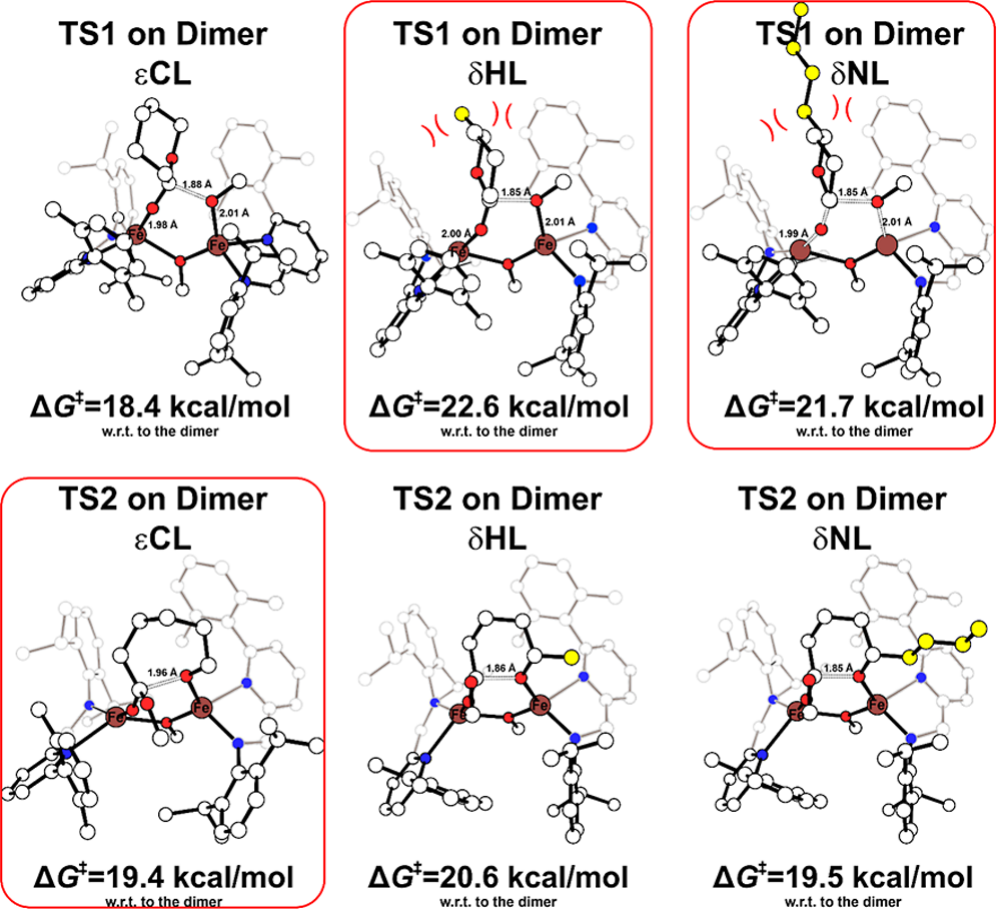
DFT-optimized structures and Gibbs energies (kcal/mol) for nucleophilic attack (TS1) and ring-opening steps TSs (TS2) for the initiation ROP of different monomers promoted by dimeric complexes. Distances are reported in Å, H atoms are omitted for clarity, and RDS is circled in red.

The high energetic stability of the dimeric alkoxide intermediate reported in Figure 4 leads us to search for an alternative mechanism that bypasses this bottleneck (Mechanism C in Figure 4), which, at least during the initiation step, does not involve the formation of MOX. In this pathway, we propose a novel TS, in which the nucleophilic attack and the departure of the amide group occur simultaneously in a concerted manner. The optimized structures for εCL, δHL, and δNL are shown in Figure S13 and are quite similar. Interestingly, the energetics we computed for Mechanism C are in the same order of magnitude as the initiation paths of monomeric species reported in Figures 5 and S11, making it a valuable working hypothesis.

Once we assessed the (possible) initiation paths, we wanted to achieve insights into the polymerization paths. Indeed, an increase in repulsive contributions arising from the interaction between the monomer’s side pendant group and the growing polymer chain is expected moving from εCL to δNL as found in organocatalysis.^13^ Since this repulsion is associated with monomer–chain interactions, it should be only marginally affected by the specific nature of the active site. So, we modeled the propagation steps of εCL, δHL, and δNL at the monomeric Fe­(II) species following Mechanism A^58^ further supported by our recent findings on the similar structural features reported by ROP of dinuclear and mononuclear zinc β-diiminate complexes.^59^

The comparison of the DFT-optimized structures and Gibbs energies for ROP propagation based on nucleophilic addition (TS1′) and ring opening (TS2′) steps is reported in Figure 7. We remark on the switch of RDS moving from initiation to propagation steps (RDS is TS1′ for εCL and TS2′ for δHL and δNL), a feature already reported in literature for ROP of lactide.^60,61^ By using the εCL as a reference point, DFT calculations predict a ΔΔ*G* ^‡^_calc_ difference of 1.4 and 3.8 kcal/mol for RDSs of δHL and δNL (Figure 7), in good agreement with the experimental *k*_app_ ratio (Figure 2) summarized in Table S7.

**Figure 7:**
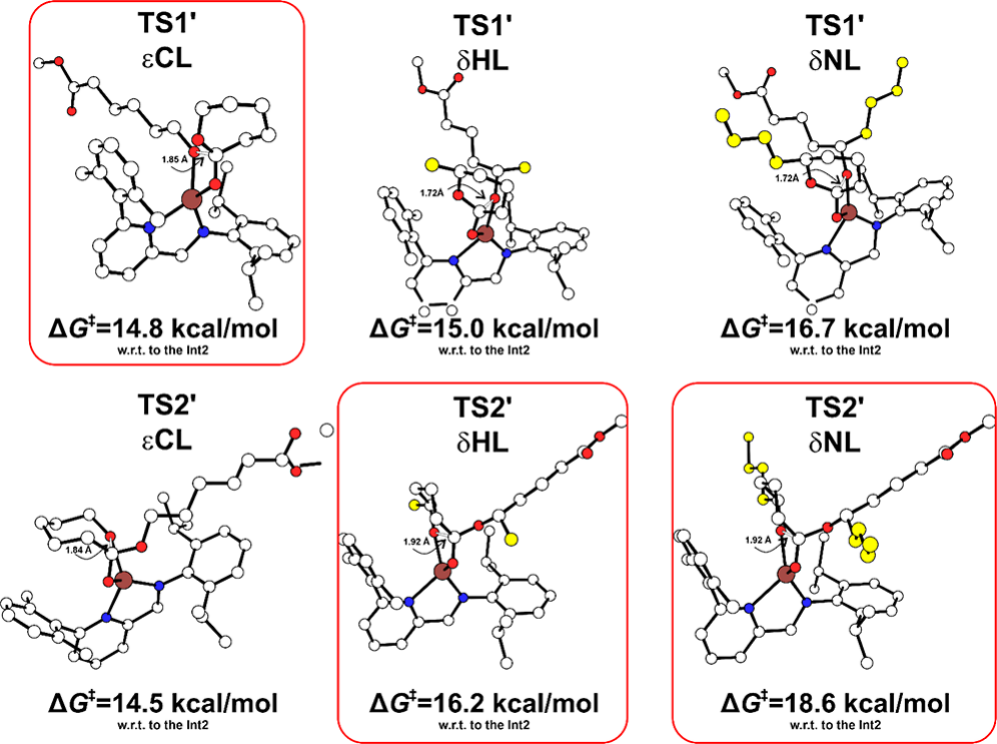
DFT-optimized structures and Gibbs energies (kcal/mol) for the nucleophilic attack (TS1′) and the ring-opening step (TS2′) for propagation ROP of εCL, δHL, and δNL, respectively. The activation energies are reported with respect to the Int2 (in kcal/mol). Distances are reported in Å, H atoms are omitted for clarity, and RDS is circled in red.

The reliability of our computational methodology for the MEPs has also been evaluated through a benchmark analysis involving alternative functionals applied to selected critical points (including TSs and intermediates). The consistency of reaction profiles across methods confirms the robustness of the proposed mechanism (Table S8 and further details can be found in Supporting Information).

To deepen this difference for the two δ-alkyl−δ-lactones and εCL, an analysis of the monomer–chain interactions on TS2′ has been performed with a noncovalent interactions (NCIs) analysis, using NCIPLOT^46^ software and visualized by VMD.^47^ NCI analysis is providing notable insights into the field of stereoselective ROP, catalyzed by both metal catalysts and organocatalysts.^62–65^ Notwithstanding that these weak interactions are generally not designed on purpose, they are showing the potential ability to modulate the catalytic process, highlighting the urgency of a better understanding of their occurrence in order to finely tune the catalyst-ligand geometry and steric/electronic features to enhance their effects. The qualitative promolecular analysis of δHL (Figure 8a) and δNL (Figure 8b) RDSs revealed a quasi-repulsive interaction between the side chain of the growing chain and the growing chain itself. This interaction appears to be more pronounced in δNL than in δHL, as highlighted in the red boxes.

**Figure 8:**
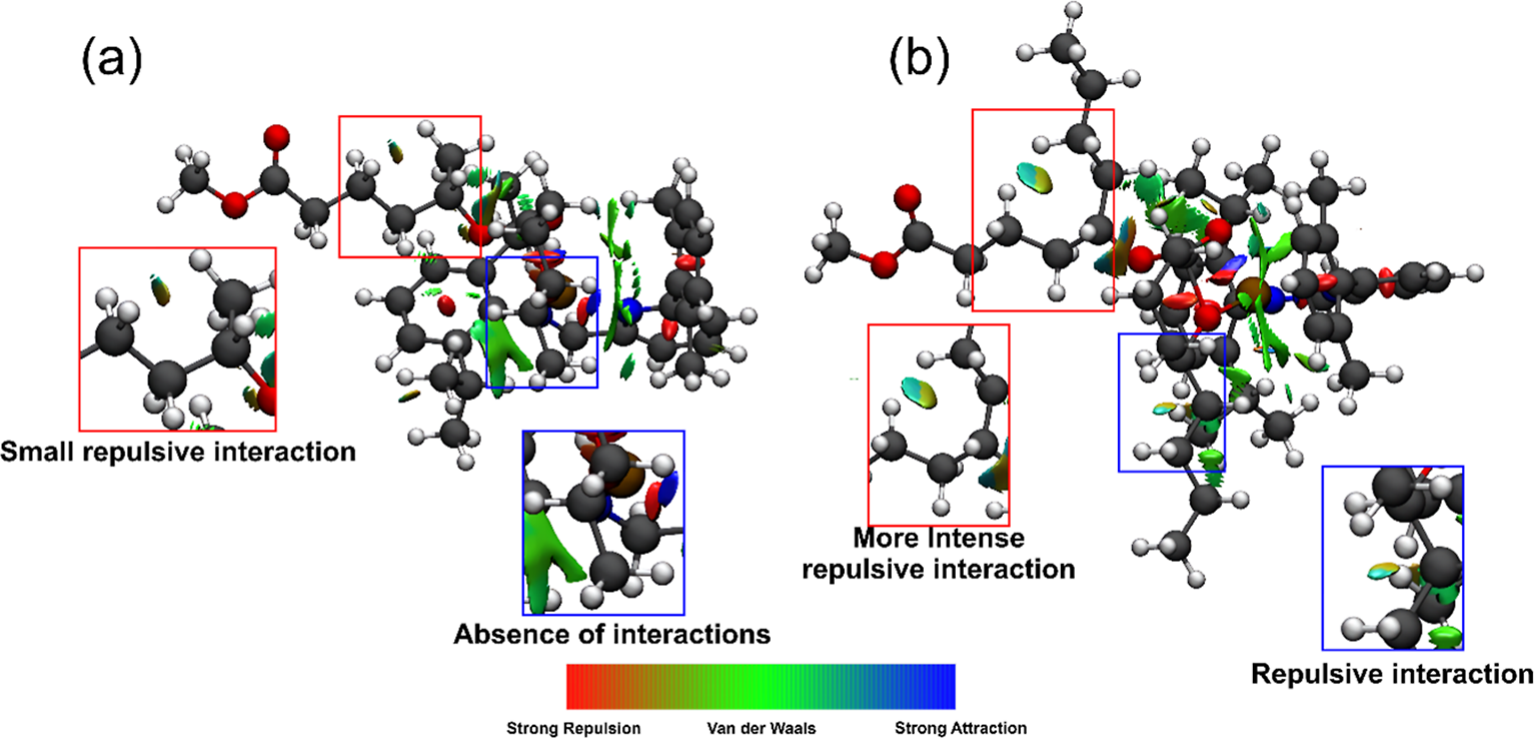
NCI analysis on TS2′ structures of δHL (a) and δNL (b). The NCI surface corresponds to *s* = 0.3 au and a color scale of −3 < ρ < 3 au for promolecular densities.

## Conclusions

In this work, we investigated the ROP of naturally occurring ε- and δ- lactones using iron-based catalysts through an integrated experimental and computational approach. The catalytic system demonstrated high activity, with turnover frequencies (TOFs) among the highest reported for δ-substituted δ-lactones, underscoring its efficiency. Kinetic analyses of ε-caprolactone, ε-decalactone, and a series of δ-lactones (δ-hexalactone, δ-nonalactone, and δ-undecalactone) revealed tunable propagation rate constants (*k*_app_), enabling precise control over copolymer architectures. This opens the route for tuning molecular architectures based on block copolymers, and diblock and triblock copolymers using HL as a soft block segment and polylactide as hard blocks were successfully synthesized, proving a living character of the catalysts. This promising structural design will be the subject of future investigations.

DFT calculations supported the experimental findings by identifying dinuclear iron complexes as plausible active species and elucidating how the monomer structure governs the rate-determining steps. The good agreement between computational predictions and experimental observations lends confidence to the proposed mechanistic interpretation. Motivated by this mechanistic understanding, preliminary calculations indicate that the catalytic activity in the ROP of δ- and ε-lactones strongly depends on the divalent metal. Activity increases from Zn (d^10^)^66^ to Fe (d^6^), likely due to the higher electron deficiency of Fe. The d-orbital configuration also affects metal size, modulating the space for monomer coordination within the catalytic pocket and, ultimately, the overall activity.^67^ Further studies on Ca (d^0^) are ongoing to explore this effect. Overall, this work highlights the potential of earth-abundant metal catalysts^68^ in the design of efficient and sustainable ROP processes for sustainable materials.^69^

## Supplementary Material





## Notes

Cartesian coordinates of the structures discussed (XYZ)
Comparison with literature data, thermal analysis, further experimental investigations, and computational details (PDF)

## References

[1] Vidal F., van der Marel E. R., Kerr R. W. F., McElroy C., Schroeder N., Mitchell C., Rosetto G., Chen T. T. D., Bailey R. M., Hepburn C., Redgwell C., Williams C. K. (2024). Designing a Circular
Carbon and Plastics Economy for a Sustainable Future. Nature.

[2] Shi C., Quinn E. C., Diment W. T., Chen E. Y.-X. (2024). Recyclable and
(Bio)­Degradable Polyesters in a Circular Plastics Economy. Chem. Rev..

[3] Shi C., Quinn E. C., Diment W. T., Chen E. Y.-X. (2024). Correction to
Recyclable and (Bio)­Degradable Polyesters in a Circular Plastics Economy. Chem. Rev..

[4] Kim M. S., Chang H., Zheng L., Yan Q., Pfleger B. F., Klier J., Nelson K., Majumder E. L.-W., Huber G. W. (2023). A Review
of Biodegradable Plastics: Chemistry, Applications, Properties, and
Future Research Needs. Chem. Rev..

[5] Lebreton L. C. M., van der Zwet J., Damsteeg J.-W., Slat B., Andrady A., Reisser J. (2017). River Plastic
Emissions to the World’s Oceans. Nat.
Commun..

[6] Leslie H. A., van Velzen M. J. M., Brandsma S. H., Vethaak A. D., Garcia-Vallejo J. J., Lamoree M. H. (2022). Discovery and Quantification of Plastic
Particle Pollution
in Human Blood. Environ. Int..

[7] Tsuji H. (2016). Poly­(Lactic
Acid) Stereocomplexes: A Decade of Progress. Adv. Drug Delivery Rev..

[8] D’Alterio M. C., D’Auria I., Gaeta L., Tedesco C., Brenna S., Pellecchia C. (2022). Are Well Performing
Catalysts for the Ring Opening
Polymerization of L-Lactide under Mild Laboratory Conditions Suitable
for the Industrial Process? The Case of New Highly Active Zn­(II) Catalysts. Macromolecules.

[9] Coates G. W., Getzler Y. D. Y. L. (2020). Chemical Recycling to Monomer for an Ideal, Circular
Polymer Economy. Nat. Rev. Mater..

[10] Hong M., Chen E. Y.-X. (2016). Completely Recyclable Biopolymers
with Linear and Cyclic
Topologies via Ring-Opening Polymerization of γ-Butyrolactone. Nat. Chem..

[11] Zhu J.-B., Watson E. M., Tang J., Chen E. Y.-X. (2018). A Synthetic Polymer
System with Repeatable Chemical Recyclability. Science.

[12] Li C., Wang L., Yan Q., Liu F., Shen Y., Li Z. (2022). Rapid and Controlled Polymerization
of Bio-Sourced δ-Caprolactone
toward Fully Recyclable Polyesters and Thermoplastic Elastomers. Angew. Chem., Int. Ed..

[13] Schneiderman D. K., Hillmyer M. A. (2016). Aliphatic Polyester Block Polymer Design. Macromolecules.

[14] McMichael P., Schultze X., Cramail H., Peruch F. (2023). Sourcing Thermodynamics,
and Ring-Opening (Co)­Polymerization of Substituted δ-Lactones:
A Review. Polym. Chem..

[15] Save M., Soum A. (2002). Controlled Ring-Opening Polymerization of Lactones and Lactide Initiated
by Lanthanum Isopropoxide, 2. Mechanistic Studies. Macromol. Chem. Phys..

[16] Ramos-Durán G., González-Zarate A. d. C., Enríquez-Medrano F. J., Salinas-Hernández M., De Jesús-Téllez M. A., Díaz de León R., López-González H. R. (2022). Synthesis
of Copolyesters Based on Substituted and Non-Substituted Lactones
towards the Control of Their Crystallinity and Their Potential Effect
on Hydrolytic Degradation in the Design of Soft Medical Devices. RSC Adv..

[17] Bandelli D., Weber C., Schubert U. S. (2019). Strontium
Isopropoxide: A Highly
Active Catalyst for the Ring-Opening Polymerization of Lactide and
Various Lactones. Macromol. Rapid Commun..

[18] Duparc V. H., Shakaroun R. M., Slawinski M., Carpentier J.-F., Guillaume S. M. (2020). Ring-Opening (Co)­Polymerization of Six-Membered Substituted
δ-Valerolactones with Alkali Metal Alkoxides. Eur. Polym. J..

[19] Xu C., Wang L., Liu Y., Niu H., Shen Y., Li Z. (2023). Rapid and Controlled Ring-Opening
(Co)­Polymerization of Bio-Sourced
Alkyl-δ-Lactones To Produce Recyclable (Co)­Polyesters and Their
Application as Pressure-Sensitive Adhesives. Macromolecules.

[20] Li G., Lamberti M., Pappalardo D., Pellecchia C. (2012). Random Copolymerization
of ε-Caprolactone and Lactides Promoted by Pyrrolylpyridylamido
Aluminum Complexes. Macromolecules.

[21] Naddeo M., D’Auria I., Viscusi G., Gorrasi G., Pellecchia C., Pappalardo D. (2020). Tuning the Thermal Properties of Poly­(Ethylene)-like
Poly­(Esters) by Copolymerization of ε-Caprolactone with Macrolactones,
in the Presence of a Pyridylamidozinc­(II) Complex. J. Polym. Sci..

[22] D’Auria I., Ferrara V., Tedesco C., Kretschmer W., Kempe R., Pellecchia C. (2021). Guanidinate
Zn­(II) Complexes as Efficient
Catalysts for Lactide Homo- and Copolymerization under Industrially
Relevant Conditions. ACS Appl. Polym. Mater..

[23] Gravina G., Pierri G., Pellecchia C. (2024). New Highly
Active Fe­(II) Pyridylamido
Catalysts for the Ring Opening Polymerization and Copolymerization
of Cyclic Esters. Mol. Catal..

[24] Wang Y., Wang X., Zhang W., Sun W.-H. (2023). Progress
of Ring-Opening
Polymerization of Cyclic Esters Catalyzed by Iron Compounds. Organometallics.

[25] Rittinghaus R. D., Schäfer P. M., Albrecht P., Conrads C., Hoffmann A., Ksiazkiewicz A. N., Bienemann O., Pich A., Herres-Pawlis S. (2019). New Kids in
Lactide Polymerization: Highly Active and Robust Iron Guanidine Complexes
as Superior Catalysts. ChemSusChem.

[26] Gibson V. C., Marshall E. L., Navarro-Llobet D., White A. J. P., Williams D. J. (2002). A Well-Defined
Iron­(Ii) Alkoxide Initiator for the Controlled Polymerisation of Lactide. J. Chem. Soc., Dalton Trans..

[27] Hashmi O. H., Capet F., Visseaux M., Champouret Y. (2022). Homoleptic
and Heteroleptic Substituted Amidomethylpyridine Iron Complexes: Synthesis,
Structure and Polymerization of Rac-Lactide. Eur. J. Inorg. Chem..

[28] Hillmyer M. A., Tolman W. B. (2014). Aliphatic Polyester Block Polymers: Renewable, Degradable,
and Sustainable. Acc. Chem. Res..

[29] D’Auria I., D’Alterio M. C., Talarico G., Pellecchia C. (2018). Alternating
Copolymerization of CO_2_ and Cyclohexene Oxide by New Pyridylamidozinc­(II)
Catalysts. Macromolecules.

[30] Frisch, M. J. ; Trucks, G. W. ; Schlegel, H. B. ; Scuseria, G. E. ; Robb, M. A. ; Cheeseman, J. R. ; Scalmani, G. ; Barone, V. ; Petersson, G. A. ; Nakatsuji, H. ; Li, X. ; Caricato, M. ; Marenich, A. V. ; Bloino, J. ; Janesko, B. G. ; Gomperts, R. ; Mennucci, B. ; Hratchian, H. P. ; Ortiz, J. V. ; Izmaylov, A. F. ; Sonnenberg, J. L. ; Williams, D. F. ; Lipparini, F. ; Egidi, F. ; Goings, J. ; Peng, B. ; Petrone, A. ; Henderson, T. ; Ranasinghe, D. ; Zakrzewski, V. G. ; Gao, J. ; Rega, N. ; Zheng, G. ; Liang, W. ; Hada, M. ; Ehara, M. ; Toyota, K. ; Fukuda, R. ; Hasegawa, J. ; Ishida, M. ; Nakajima, T. ; Honda, Y. ; Kitao, O. ; Nakai, H. ; Vreven, T. ; Throssell, K. ; Montgomery, J. A., Jr. ; Peralta, J. E. ; Ogliaro, F. ; Bearpark, M. J. ; Heyd, J. J. ; Brothers, E. N. ; Kudin, K. N. ; Staroverov, V. N. ; Keith, T. A. ; Kobayashi, R. ; Normand, J. ; Raghavachari, K. ; Rendell, A. P. ; Burant, J. C. ; Iyengar, S. S. ; Tomasi, J. ; Cossi, M. ; Millam, J. M. ; Klene, M. ; Adamo, C. ; Cammi, R. ; Ochterski, J. W. ; Martin, R. L. ; Morokuma, K. ; Farkas, O. ; Foresman, J. B. ; Fox, D. J. Gaussian 16, Revision C.01: Wallingford, CT, 2016.

[31] Becke A. D. (1988). Density-Functional
Exchange-Energy Approximation with Correct Asymptotic Behavior. Phys. Rev. A.

[32] Lee C., Yang W., Parr R. G. (1988). Development
of the Colle-Salvetti
Correlation-Energy Formula into a Functional of the Electron Density. Phys. Rev. B.

[33] Hay P. J., Wadt W. R. (1985). Ab Initio Effective
Core Potentials for Molecular Calculations.
Potentials for K to Au Including the Outermost Core Orbitals. J. Chem. Phys..

[34] Schäfer A., Horn H., Ahlrichs R. (1992). Fully Optimized
Contracted Gaussian
Basis Sets for Atoms Li to Kr. J. Chem. Phys..

[35] Cossi M., Barone V., Cammi R., Tomasi J. (1996). Ab Initio Study of
Solvated Molecules: A New Implementation of the Polarizable Continuum
Model. Chem. Phys. Lett..

[36] Grimme S., Ehrlich S., Goerigk L. (2011). Effect of the Damping
Function in
Dispersion Corrected Density Functional Theory. J. Comput. Chem..

[37] Wadt W. R., Hay P. J. (1985). Ab Initio Effective
Core Potentials for Molecular Calculations.
Potentials for Main Group Elements Na to Bi. J. Chem. Phys..

[38] McLean A. D., Chandler G. S. (1980). Contracted Gaussian
Basis Sets for Molecular Calculations.
I. Second Row Atoms, Z = 11–18. J. Comput.
Phys..

[39] Krishnan R., Binkley J. S., Seeger R., Pople J. A. (1980). Self-consistent
Molecular Orbital Methods. XX. A Basis Set for Correlated Wave Functions. J. Chem. Phys..

[40] Zhao Y., Truhlar D. G. (2008). The M06 Suite of
Density Functionals for Main Group
Thermochemistry, Thermochemical Kinetics, Noncovalent Interactions,
Excited States, and Transition Elements: Two New Functionals and Systematic
Testing of Four M06-Class Functionals and 12 Other Functionals. Theor. Chem. Acc..

[41] Adamo C., Barone V. (1999). Toward Reliable Density
Functional Methods without
Adjustable Parameters: The PBE0Model. J. Chem.
Phys..

[42] Ernzerhof M., Scuseria G. E. (1999). Assessment of the
Perdew–Burke–Ernzerhof
Exchange-Correlation Functional. J. Chem. Phys..

[43] Tao J., Perdew J. P., Staroverov V. N., Scuseria G. E. (2003). Climbing the Density
Functional Ladder: Nonempirical Meta–Generalized Gradient Approximation
Designed for Molecules and Solids. Phys. Rev.
Lett..

[44] Chai J.-D., Head-Gordon M. (2008). Long-Range
Corrected Hybrid Density Functionals with
Damped Atom–Atom Dispersion Corrections. Phys. Chem. Chem. Phys..

[45] Falivene L., Barone V., Talarico G. (2018). Unraveling
the Role of Entropy in
Tuning Unimolecular vs. Bimolecular Reaction Rates: The Case of Olefin
Polymerization Catalyzed by Transition Metals. Mol. Catal..

[46] Boto R. A., Peccati F., Laplaza R., Quan C., Carbone A., Piquemal J.-P., Maday Y., Contreras-García J. (2020). NCIPLOT4:
Fast, Robust, and Quantitative Analysis of Noncovalent Interactions. J. Chem. Theory Comput..

[47] Humphrey W., Dalke A., Schulten K. V. M. D. (1996). VMD: Visual molecular dynamics. J. Mol. Graph..

[48] Pan Y., Hao M., Li X., Meng Y., Kang X., Zhang G., Sun X., Song X.-Z., Zhang L., So Y.-M. (2025). Anilido-Oxazoline-Ligated
Iron Alkoxide Complexes for Living Ring-Opening Polymerization of
Cyclic Esters with Controllability. Inorg. Chem..

[49] Li Z., Shen Y., Li Z. (2024). Ring-Opening
Polymerization of Lactones
to Prepare Closed-Loop Recyclable Polyesters. Macromolecules.

[50] Falivene L., Cao Z., Petta A., Serra L., Poater A., Oliva R., Scarano V., Cavallo L. (2019). Towards the Online Computer-Aided
Design of Catalytic Pockets. Nat. Chem..

[51] Falivene L., Cavallo L., Talarico G. (2015). Buried Volume
Analysis for Propene
Polymerization Catalysis Promoted by Group 4 Metals: A Tool for Molecular
Mass Prediction. ACS Catal..

[52] Cicolella A., Romano E., Barone V., De Rosa C., Talarico G. (2022). Metallocenes
and Beyond for Propene Polymerization: Energy Decomposition of Density
Functional Computations Unravels the Different Interplay of Stereoelectronic
Effects. Organometallics.

[53] Romano E., Barone V., Budzelaar P. H. M., De Rosa C., Talarico G. (2024). Revisiting
Stereoselective Propene Polymerization Mechanisms: Insights through
the Activation Strain Model. Chem.—Asian
J..

[54] D’Alterio M. C., Moccia S., Rusconi Y., De Rosa C., Talarico G. (2024). Ligand Coordination
Controlled by Monomer Binding: A Hint from DFT for Stereoselective
Lactide Polymerization. Catal. Sci. Technol..

[55] Moccia S., D’ Alterio M. C., Romano E., De Rosa C., Talarico G. (2025). Stereoselectivity
Control Interplay in Racemic Lactide Polymerization by Achiral Al-Salen
Complexes. Macromol. Rapid Commun..

[56] Poater A., Cavallo L. (2009). Comparing Families of Olefin Polymerization Precatalysts
Using the Percentage of Buried Volume. Dalton
Trans..

[57] Escayola S., Bahri-Laleh N., Poater A. (2024). %VBur Index and Steric Maps: From
Predictive Catalysis to Machine Learning. Chem.
Soc. Rev..

[58] We Are Fully Aware That, to More Accurately Reflect the Real System, the Propagation Step Should Ideally Be Modeled at the Level of the Dimeric Species (MOX)2. However, the Nonet Spin Multiplicity of This Species Makes Such Modeling Computationally Prohibitive. Nonetheless, It Is Reasonable to Expect That Any Differences Observed between the Two Monomers during the Propagation Step Would Persist, or Even Become More Pronounced, in the Corresponding Dimeric Species.

[59] Rusconi Y., D’Alterio M. C., De Rosa C., Coates G. W., Talarico G. (2025). Disclosing
Multiple Factors Influencing Enantioselective Copolymerization of
CO2 with Meso-Epoxides Using β-Diiminate Zn Catalysts. Green Chem..

[60] D’Alterio M. C., De Rosa C., Talarico G. (2020). Stereoselective
Lactide Polymerization:
The Challenge of Chiral Catalyst Recognition. ACS Catal..

[61] Rusconi Y., D’Alterio M. C., De Rosa C., Lu Y., Severson S. M., Coates G. W., Talarico G. (2024). Mechanism of Alternating Poly­(Lactic-Co-Glycolic
Acid) Formation by Polymerization of (S)- and (R)-3-Methyl Glycolide
Using an Enantiopure Aluminum Complex. ACS Catal..

[62] Gesslbauer S., Savela R., Chen Y., White A. J. P., Romain C. (2019). Exploiting
Noncovalent Interactions for Room-Temperature Heteroselective Rac-Lactide
Polymerization Using Aluminum Catalysts. ACS
Catal..

[63] Fosu S. A., Vlaisavljevich B. (2025). Stereoselective
Ring Opening Polymerization of Lactide
Using Chiral Aluminum Salan Catalysts. Dalton
Trans..

[64] Falivene L., Cavallo L., Talarico G. (2020). The Role of Noncovalent Interactions
in Olefin Polymerization Catalysis: A Further Look to the Fluorinated
Ligand Effect. Mol. Catal..

[65] Zunino R., Falivene L., Talarico G. (2025). Looking into the Quest for Stereoselective
Ring-Opening Polymerization of Racemic Lactide with Chiral Organocatalysts. ACS Catal..

[66] D’Auria I., D’Alterio M. C., Tedesco C., Pellecchia C. (2019). Tailor-Made
Block Copolymers of l-, d- and Rac-Lactides and ε-Caprolactone
via One-Pot Sequential Ring Opening Polymerization by Pyridylamidozinc­(Ii)
Catalysts. RSC Adv..

[67] Rusconi Y., D’Alterio M. C., Grillo A., Poater A., De Rosa C., Talarico G. (2024). The Metal Role on the Activity and Stereoselectivity
of Ring Opening Polymerization of Racemic Lactide Promoted by Salen
Catalysts. Polymer.

[68] Rittinghaus R. D., Herres-Pawlis S. (2023). Catalysts as Key Enablers for the Synthesis of Bioplastics
with Sophisticated Architectures. Chem.—Eur.
J..

[69] Han J. W., Hollmann F., Luque R., Song I. K., Talarico G., Tatsumi T., Yan N. (2022). Molecular Catalysis
for the Chemistry
of the Future: A Perspective. Mol. Catal..

