# Supplementary material for: Sustainable Polymerization of Natural Lactones via Iron Catalysis: An Integrated Experimental and Computational Study
Source: ACS Sustain Chem Eng. 2025 Nov 5;13(45):19613–24. doi: 10.1021/acssuschemeng.5c07947 (PMC12628644; doi:10.1021/acssuschemeng.5c07947)
Supplement: Supplementary file 2 [file sc5c07947_si_002.pdf]

Supporting Information of

Sustainable Polymerization of Natural Lactones via Iron Catalysis: An Integrated Experimental and Computational Study

Giuseppe Gravina,[1] Eugenio Romano,[2,3,4] Alessia Liporace,[1] Massimo Christian D'Alterio,[2] Giovanni Talarico,[2,3] Claudio Pellecchia[1]**

[1] Dipartimento di Chimica e Biologia "A. Zambelli", Università degli Studi di Salerno, via Giovanni Paolo II 132, Fisciano, SA 84084, Italy.
[2] Dipartimento di Scienze Chimiche, Università degli Studi di Napoli Federico II, Italy.
[3] Scuola Superiore Meridionale, Largo San Marcellino 10, Napoli 80138, Italy.
[4] Scuola Normale Superiore, Piazza dei Cavalieri 7, Pisa, 56126.
Email: cpellecchia@unisa.it; talarico@unina.it

Table of Contents

Comparison with literature data…………S2
Table S1…………S3
Table S2…………S3
Figure S1…………S4
Figure S2…………S4
Figure S3…………S5
Figure S4…………S5
Figure S5…………S6
Figure S6…………S6
Figure S7…………S7
Figure S8…………S7
Thermal analysis Table S3…………S8
Scheme S1…………S8
Scheme S2…………S8
Computational Details…………S9
Table S4…………S10
Scheme S3…………S11
Table S5…………S11
Figure S9…………S12
Figure S10…………S12
Figure S11…………S13
Table S6…………S13
Figure S12…………S14
Table S7…………S15
Figure S13…………S15
Table S8…………S16
References…………S17

Comparison with literature data

In **Table S1** we report a comparative analysis of various catalysts reported in literature. Sr(O^{i}Pr) (run 1) showed moderate activity in toluene although it yields low molecular weights polymers. Yttrium (run 2) and Lanthanum (run 3) precursors were employed in polymerization reactions characterized by extended durations and low efficiencies. Organic bases such as Triazabicyclodecene (TBD) (run 4) and Diphenyl Phosphate (DPP) (run 5) were used in the ROP of δHL in toluene solution with low TOFs and molecular weights. Methoxy systems like KOMe were used efficiently in the polymerization of δHL, especially when combined with urea in a bicomponent catalytic system. With this system Li et. al reported high molecular weights polymers. The iron catalyst reported in this work represents the first coordination complex active in the polymerization of δ-alkyl-δ-lactones, achieving good activity and control over the polymerization process in both bulk and solution phases across various temperatures. Notably, it has enabled the synthesis of high molecular weight polymers, reaching up to 50 kDa.

Table S1. Comparison with catalysts studied in the literature for the polymerization of δHL

Run[a]	Cat.	[M]:[Cat]:[ROH]	Solv.	C (M)	*T* (°C)	*t*	Conv[b] (%)	TOF (h^{-1})	M_{nThe}[c] (kDa)	$M_{n,GPC}$[e] (kDa)	*Đ*
1	$Sr(O^{i}Pr)$[1]	110:1:1	Tol	4	30	15m	82	360	3.2	5.0	1.2
2	$Y(OTf)_3$[2]	100:1:3	-	9.1	21	18h	0	-	-	-	-
					60	72h	26	0.4	-	-	-
3	$La(N(TMS)_2)_3$[2]	100:1:3	-	9.1	21	18h	0	-	-	-	-
					60	72h	44	0.6	-	-	-
4	TBD[3]	200:4:1	Tol	4	29	2h	44	11	10	6	1.1
5	DPP[4]	40:1:1	Tol	3.5	RT	24h	85	1.4	3.9	8.8	1.2
6	KOMe[5]	100:1	THF	6	29	2h	58	29	6.6	9.0	1.4
7	KOMe/Urea[5]	100:(1:1)	THF	6		10m	86	516	9.8	12.1	1.1
8	Fe(II)	400:1:1	-	9.1	80	12m	67	1600	31.0	11.7	1.6
9	Fe(II)	100:1:1	Tol	2	30	30m	64	128	8.1	15.7	1.4
10	Fe(II)	400:1:1	Tol	4	30	3h	80	73	37.0	26.7	1.1

Table S2. ROP of δHL promoted by [Fe] = 5 mM; [δHL]/[Fe]/[BnOH] = 100:1:1; *T* = 30 °C; [δHL] = 2M in toluene.

Time (min)	Conv (%)	M_n, Theo (KDa)	M_n, NMR (KDa)	M_n, GPC (KDa)	Đ
4	2	0.2	0.6	-	-
8	12	1.4	1.4	-	-
12	26	3.0	2.4	-	-
16	42	4.8	5.5	-	-
20	57	6.5	6.2	-	-
24	61	7.0	7.5	-	-
30	66	7.5	7.4	-	-
35	67	7.6	8.0	15.7	1.3

^{1}H NMR analysis

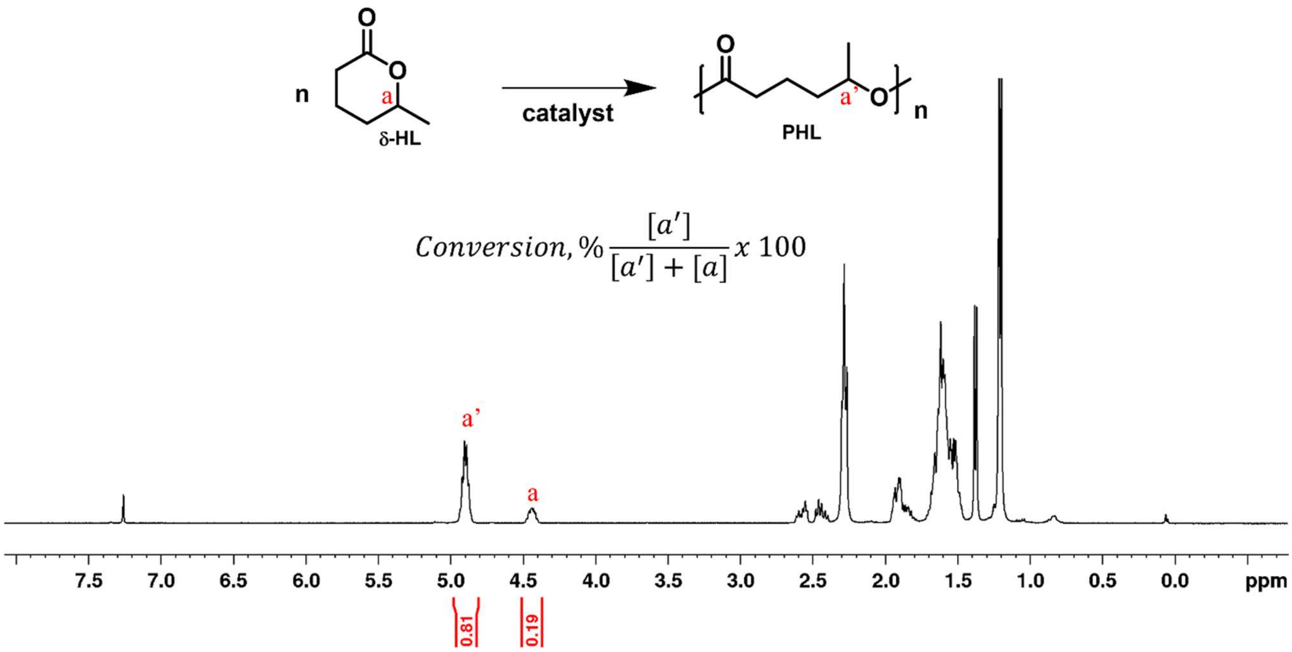

Figure S1. ^{1}H NMR spectrum (400 MHz, $CDCl_3$, 298 K) of crude material for δ-hexalactone conversion determination.

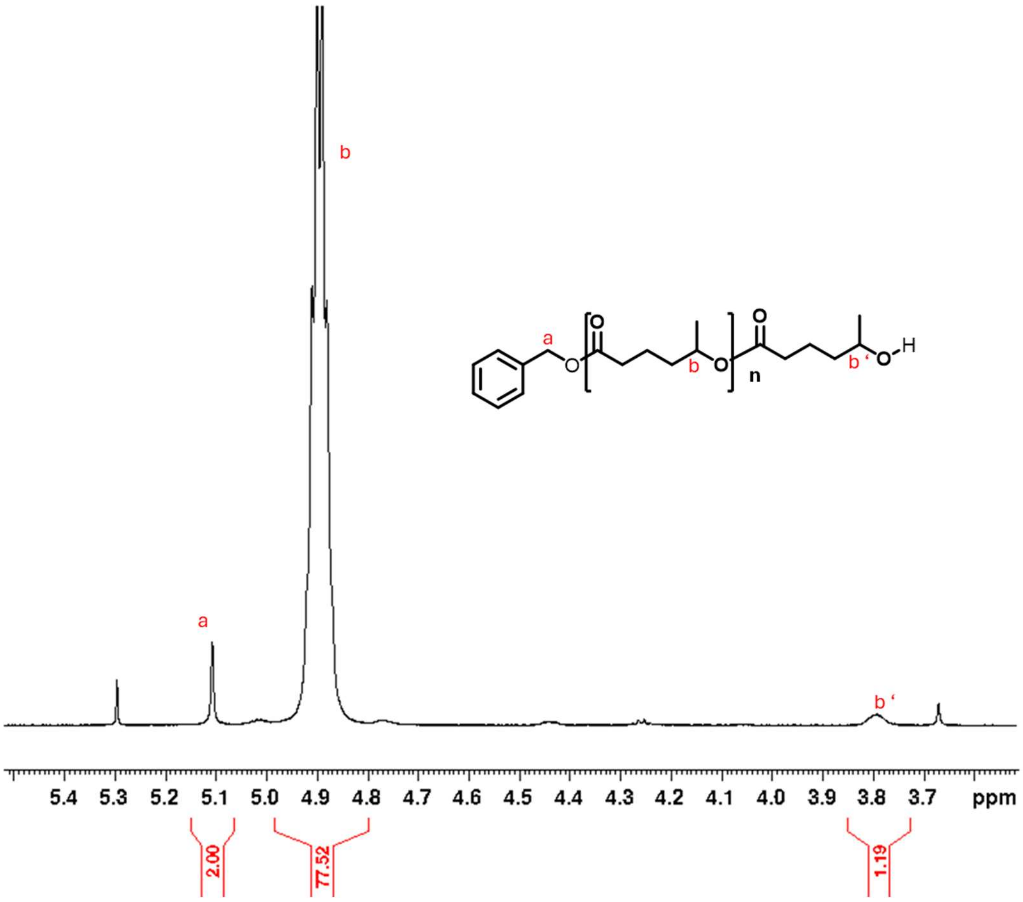

Figure S2. ^{1}H NMR spectrum (400 MHz, $CDCl_3$, 298 K) of δ-hexalactone for end-groups determination.

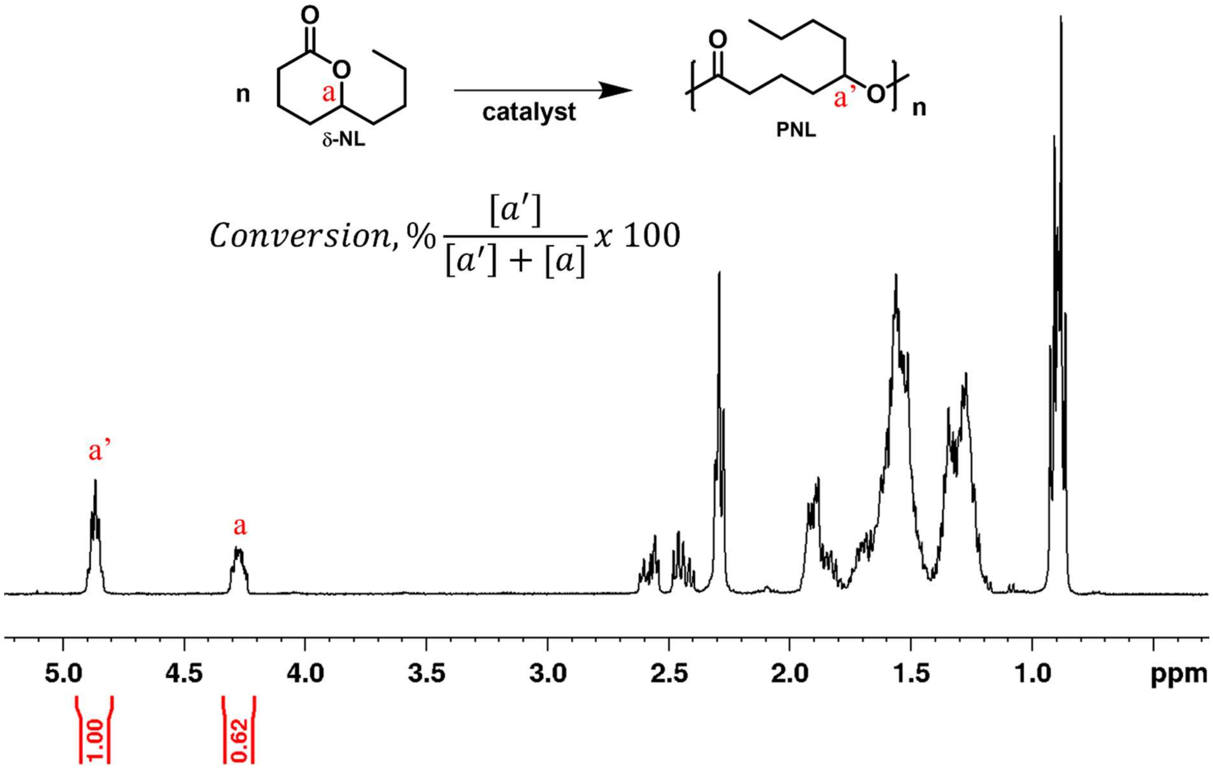

Figure S3. ^{1}H NMR spectrum (400 MHz, $CDCl_3$, 298 K) of crude material for δ-nonalactone conversion determination

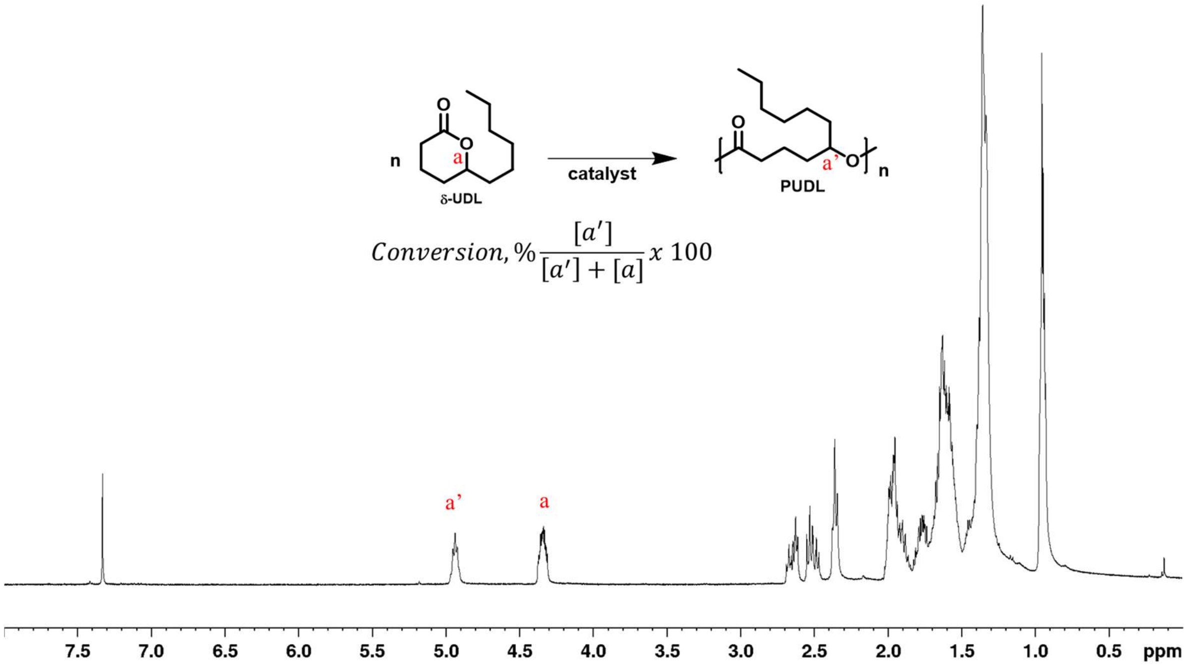

Figure S4. ^{1}H NMR spectrum (400 MHz, $CDCl_3$, 298 K) of crude material for δ-undecalactone conversion determination.

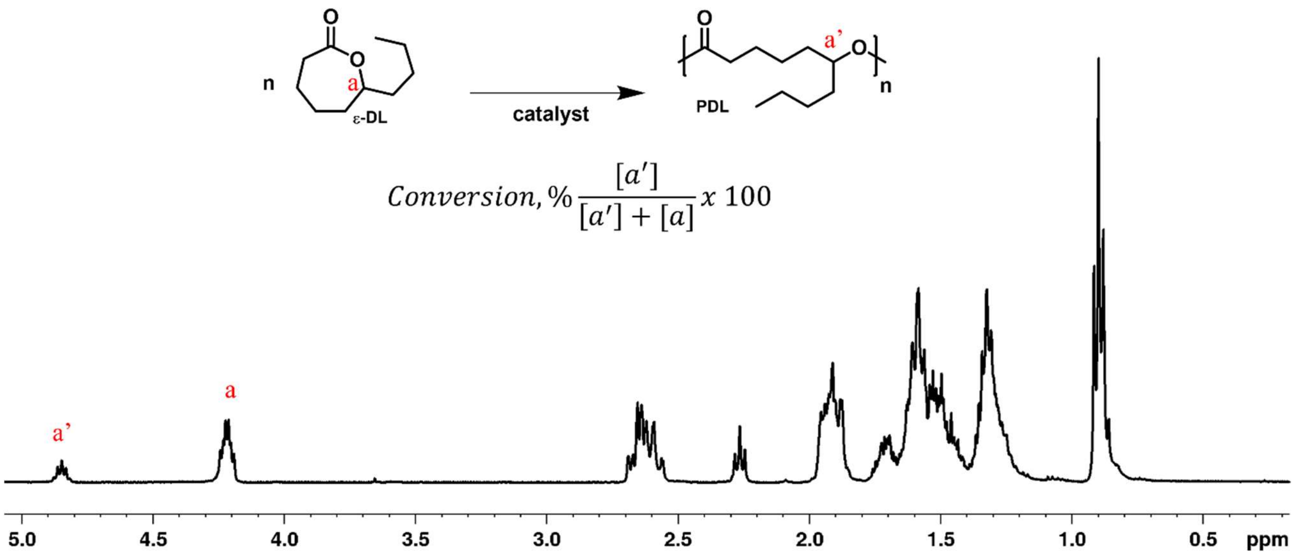

Figure S5. ^{1}H NMR spectrum (400 MHz, $CDCl_3$, 298 K) of crude material for ε-decalactone conversion determination.

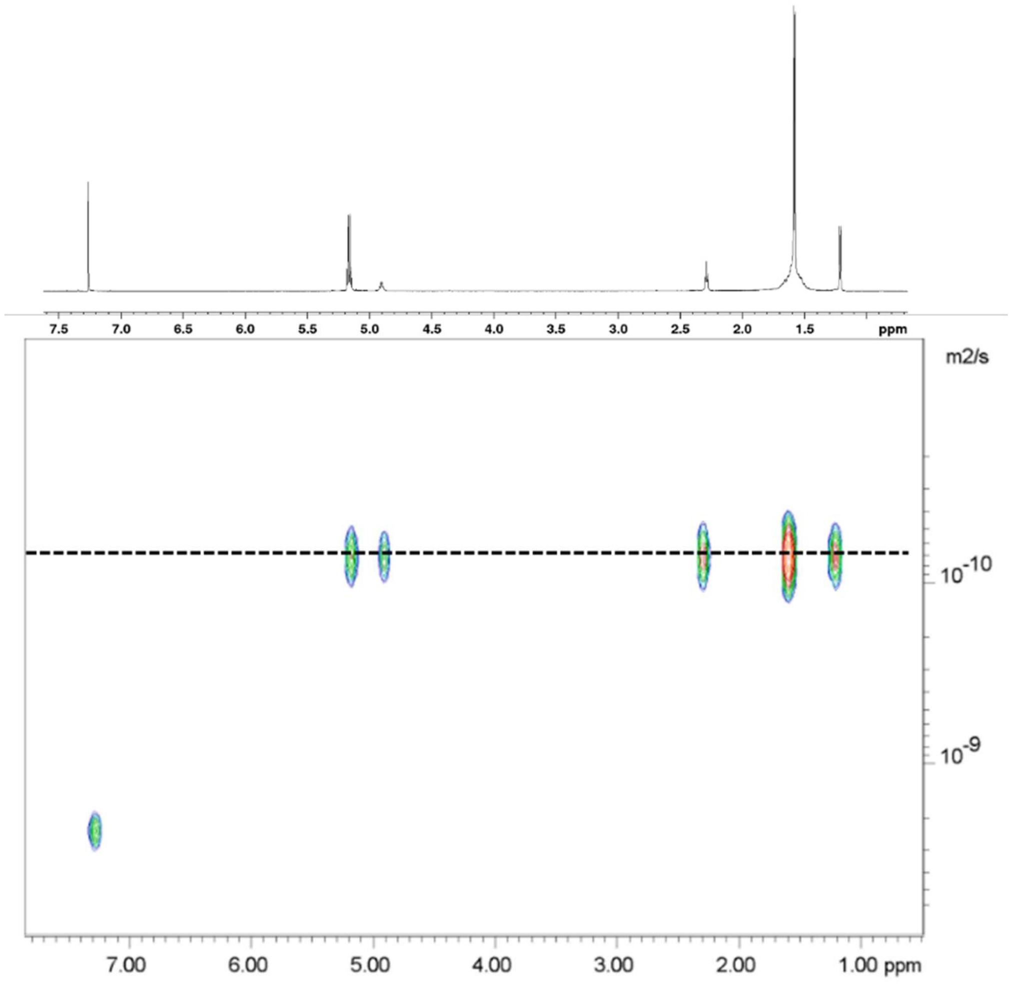

Figure S6. 2D NMR- and DOSY Spectrum of PLLA-*b*-PHL-*b*-PLLA triblock sample (Sample 2 in Table 3 of the main text).

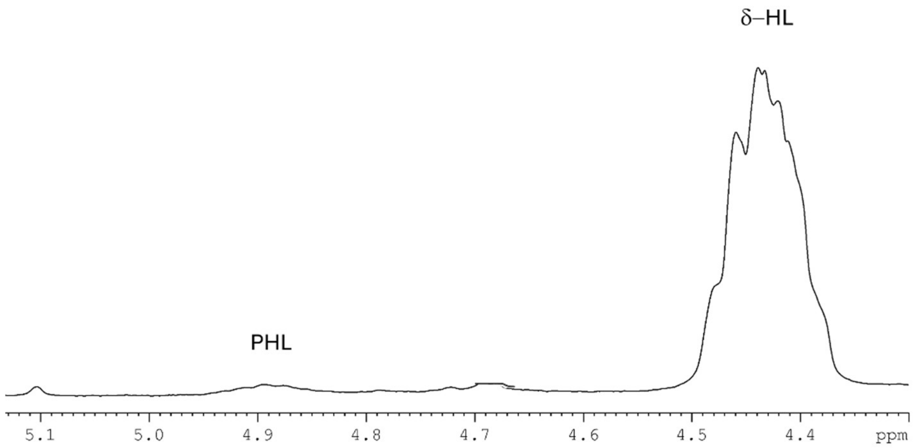

Figure S7: ^{1}H NMR spectrum (300 MHz, $CDCl_3$, 25 °C) of a depolymerization reaction mixture of poly- δ-hexalactone

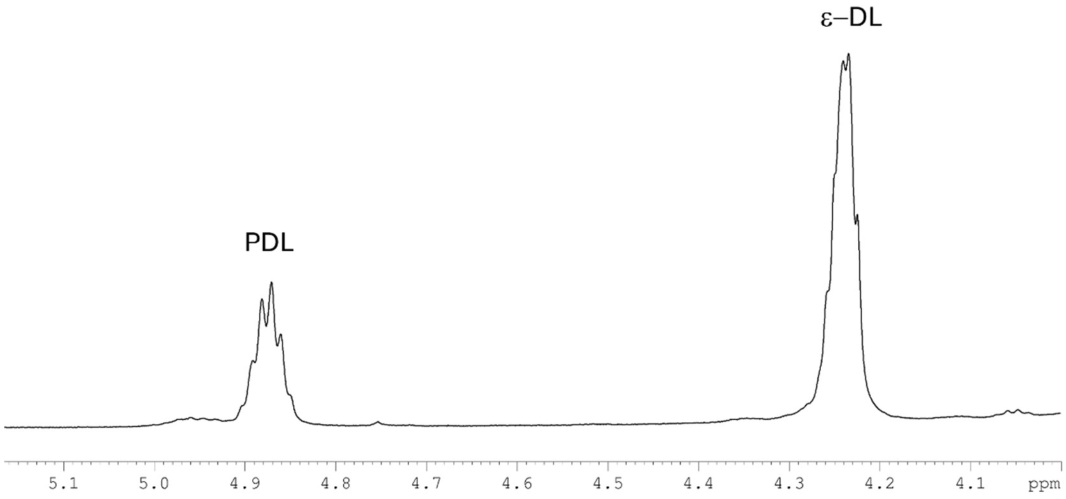

Figure S8: ^{1}H NMR spectrum (600 MHz, $CDCl_3$, 25 °C) of a depolymerization reaction mixture of poly-ε-decalactone

Thermal analysis

Table S3: Thermal properties of homopolymers.

Samples[a]	T_g[b] (°C)	T_m[b] (°C)	$T_{d,50\%}$[c] (°C)
Poly(δHL)	-37	44	268
Poly(δNL)	-55	/	321
Poly(δUNL)	-68	/	188
Poly(εCL)	-61	59	410
Poly(εDL)	-57	/	348

[a] Polymers with a M_n ranging from 8 kDa to 12kDa. [b] Determined by DSC in the first heating under a heating rate of 10°C min^{-1} [c] Determined by TGA a heating rate of 20°C min^{-1}

Further experimental investigations

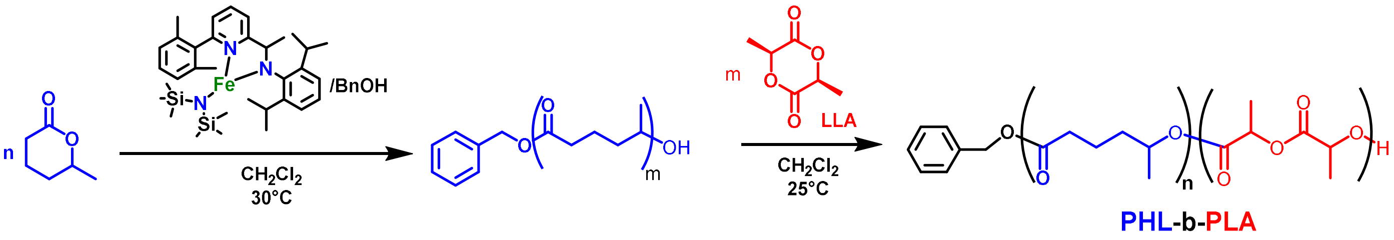

Scheme S1: Synthesis of diblock copolymer PHL-*b*-PLA.

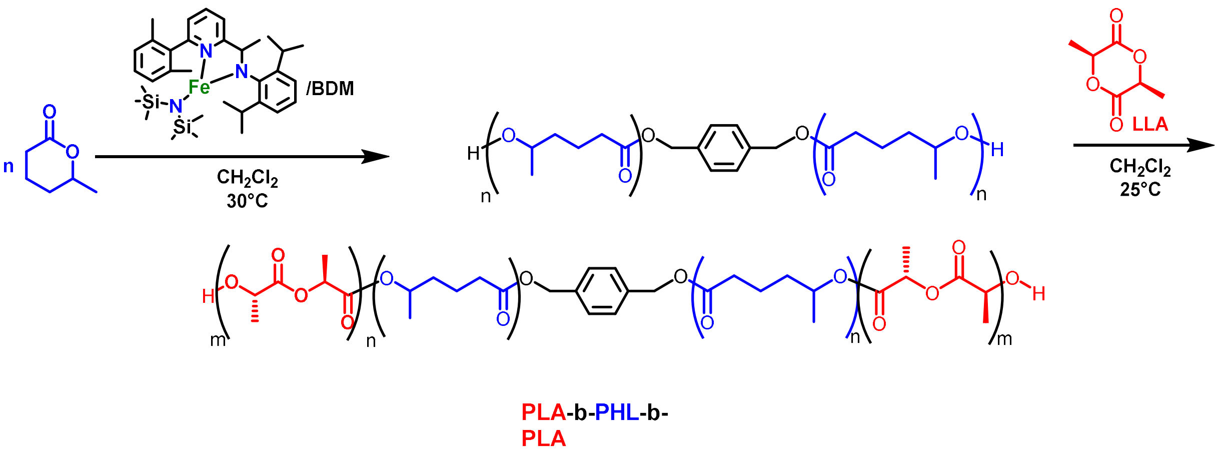

Scheme S2: Synthesis of triblock copolymer PLA-*b*-PHL-*b*-PLA.

Computational Details

Gaussian16 software[6] has been used to perform computational analysis, based on B3LYP[7,8] functional for both optimization and energy refinement steps. For the optimization, Fe atoms have been modelled using LANL2DZ[9] basis-set, including ECP[9] for core electrons, and SVP[10,11] basis set has been used for C, H, N, O, Si; in the energy refinement, solvent effects have been evaluated through PCM[12] and dichloromethane as solvent, dispersion effects have been modelled through D3BJ[13] empirical dispersion by Grimme, Fe atoms have been modelled using SDD[14] basis-set, including ECP[14] for core electrons, and 6-311g(d,p)[15,16] functional has been used to describe C, H, N, O, Si. Geometry optimizations have been carried out without symmetry constraints. Minima and transition states (TSs) have been confirmed by frequency analysis checking the presence of only one imaginary frequency for TSs. The same analysis has been employed also for the calculation of zero-point energies and thermal (enthalpy and entropy) corrections (298.15 K, 1 bar).

Theoretical investigation to evaluate the most stable spin-state on the neutral precursor, considering singlet, triplet and quintet state have been performed. The analysis revealed the quintet as the most stable and the relative values (kcal/mol), are reported in Table S4. To test the robustness of the results and support the reliability of the results obtained, the relative stability trend among spin states on B3LYP geometries has been evaluated employing several DFT functionals, spanning a broad range of theoretical approaches: meta-GGA (M06L[17] including and not including D3[18] dispersion), hybrid GGAs (B3LYP-D3BJ, PBE0[19,20] and PBE0-D3BJ), hybrid meta-GGA (M062X[17], M06[17] including and not including D3 dispersion and TPSSh[21]-D3BJ) and hybrid range-separated (ωB97X-D)[22]. Despite the differences among the functionals, such as the HF percentage (ranging from 20% to 54%, and variable in the range of 22%, short-range exact exchange, and 100%, long-range exact exchange, for ωB97X-D) and the inclusion or absence of the empirical dispersion correction (D3/D3BJ or integrated in the functionals), the trend in spin-state stability is qualitatively consistent. Indeed, the quintet state emerges as the most energetically stable, in terms of electronic energy (ΔE) and Gibbs energy (ΔG). All the results are reported in Table S4. Among the tested functional, TPSSh-D3BJ and M06L(-D3) showed excellent agreement with B3LYP-D3BJ in reproducing spin-state energetics, maintaining a balanced spin gaps. The trends have been analyzed to assess the sensitivity of spin-state energetics in Fe(II) complexes, depending on the percentage of HF exchange. Low-to-moderate HF exchange (≤20%) seems to reproduce balanced spin gaps, while increasing it over 27%, tends to overestimate the stability of high-spin state. Additionally, the spin contamination values ($\Delta\langle S^2\rangle$) for Triplet and Quintet have been reported, defined as the difference between the calculated $\langle S^2\rangle$ after spin annihilation and the expected value $S(S+1)$ ($\langle S^2\rangle_{calc.}-S(S+1)$). The values refer only to the energy refinement calculations. For the quintet state all the functionals show minimal contamination, while

for the triplet state it tends to be slightly higher, reporting B3LYP and ωB97X-D as the suggested choice to preserve a better spin purity. These results highlight the importance of functional choice when modelling transition-metal systems with multiple accessible spin-state.

A comparative benchmark with respect to the methodology reported was carried out to evaluate the robustness of this approach. Starting from the geometry optimized at B3LYP level with LANL2DZ (ECP) on Fe atoms and SVP basis set for all other atoms, energy refinement calculations were then performed using M06L[17] including D3[18] dispersion, TPSSh[21]-D3BJ and ωB97X-D[22], using SDD ECP for Fe and 6-311g(d,p) for non-metal atoms, and PCM model with dichloromethane as solvent. All computed species are in their high-spin states: quintet for mononuclear species and nonet for dinuclear species. Comparative results are reported in Table S7 and we note that B3LYP-D3BJ and TPSSh-D3BJ produced consistent activation energies across all the steps and monomer, M06L-D3 tends to slightly increase the barrier and ωB97XD, for its long-range hybrid nature, predicts lower activation barriers, especially for dinuclear species. All the functionals captured the increase in activation barrier across the monomer species: εCL<δHL<δNL, in line with the increasing of monomer steric demands.

The TSs calculations on have been performed taking into account that the nucleophilic attack by the methoxidic oxygen could occour on both *re* and *si* enantiofaces, considering only the *S* enantiomer for δHL and δNL (Scheme S1).

The Buried Volume analysis[23] of the neutral precursor was performed to quantify the steric encumbrance around the metal center. It was carried out considering a sphere 5.5 Å centered on the metal center, with a mesh of 0.1 Å and excluding the hydrogen atoms in the computation.

Additionally, BSSE correction, using Boys-Bernardi counterpoise correction,[24] on linear chain dimeric Int2 (Figure S7), has been carried out resulting in a low correction of 6.1 kcal/mol and 5.9 kcal/mol for εCL and δHL, respectively.

Table S4. Functional, type of functional, percentage of HF exchange, electronic and free energies (in kcal/mol) reported for iron(II) neutral precursor for singlet (S), triplet (T) and quintet (Q) states with respect to quintet spin state. Spin contamination values ($\Delta\langle S^2\rangle$) for Triplet and Quintet and the average for each functional have been reported.

Functional	Type	Exc. HF%	Δ*E* (S/Q)	Δ*E* (T/Q)	Δ*G* (S/Q)	Δ*G* (T/Q)	$\Delta\langle S^2\rangle$ (T)	$\Delta\langle S^2\rangle$ (Q)	Avg. $\Delta\langle S^2\rangle$
**M06L-D3**	Meta-GGA	0	42.2	18.3	45.7	19.3	0.0062	0.0002	0.0032
**M06L**	Meta-GGA	0	42.4	18.5	45.9	19.6	0.0062	0.0002	0.0032
**TPSSh-D3BJ**	Hybrid Meta-GGA	10	45.4	17.6	48.9	18.6	0.0066	0.0001	0.0034
**B3LYP-D3BJ**	Hybrid GGA	20	42.8	18.6	46.3	19.6	0.0029	0.0001	0.0015
**ωB97X-D**	Hybrid Range-sep.	22-100	48.5	22.9	52.0	23.9	0.0028	0.0000	0.0014
**PBE0-D3BJ**	Hybrid GGA	25	56.1	24.5	59.5	25.6	0.0081	0.0001	0.0041
**PBE0**	Hybrid GGA	25	59.6	26.5	63.1	27.6	0.0081	0.0001	0.0041
**M06-D3**	Hybrid Meta-GGA	27	61.9	32.3	65.4	33.4	0.0078	0.0002	0.0040
**M06**	Hybrid Meta-GGA	27	62.8	33.0	66.3	34.0	0.0078	0.0002	0.0040
**M062X**	Hybrid Meta-GGA	54	79.9	42.3	83.4	43.4	0.0059	0.0001	0.0030

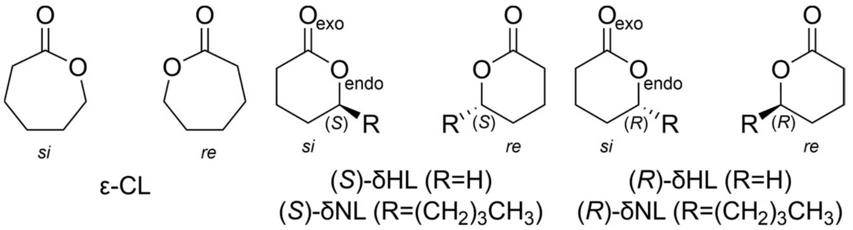

Scheme S3. Schematic representation of elements of chirality for all the monomers.

Additionally, we analyzed monomer coordination to both S_1 and S_2 coordination sites. Notwithstanding the MEP suggests a preference for the coordination at the S_2 site, the S_1 site results to be the favorite one for the coordination of the monomer with the *si* enantioface, for initiation and propagation (TS1 and TS1'). *Viceversa*, it seems that for the *re* enantioface the S_2 results to be the favorite one. In **Table 1**, TS Δ*G* values are reported for *si* enantioface.

Table S5. Free energies (Δ*G*) TS differences for εCL and δHL TS1 and TS1'. Positive values refer to preference for S_1 site.

	TS1		TS1'	
	εCL	δHL	εCL	δHL
Δ*G* (kcal/mol) *si* enantioface	0.9	1.3	0.7	2.4
Δ*G* (kcal/mol) *re* enantioface	-0.7	-1.5	0.0	-2.5

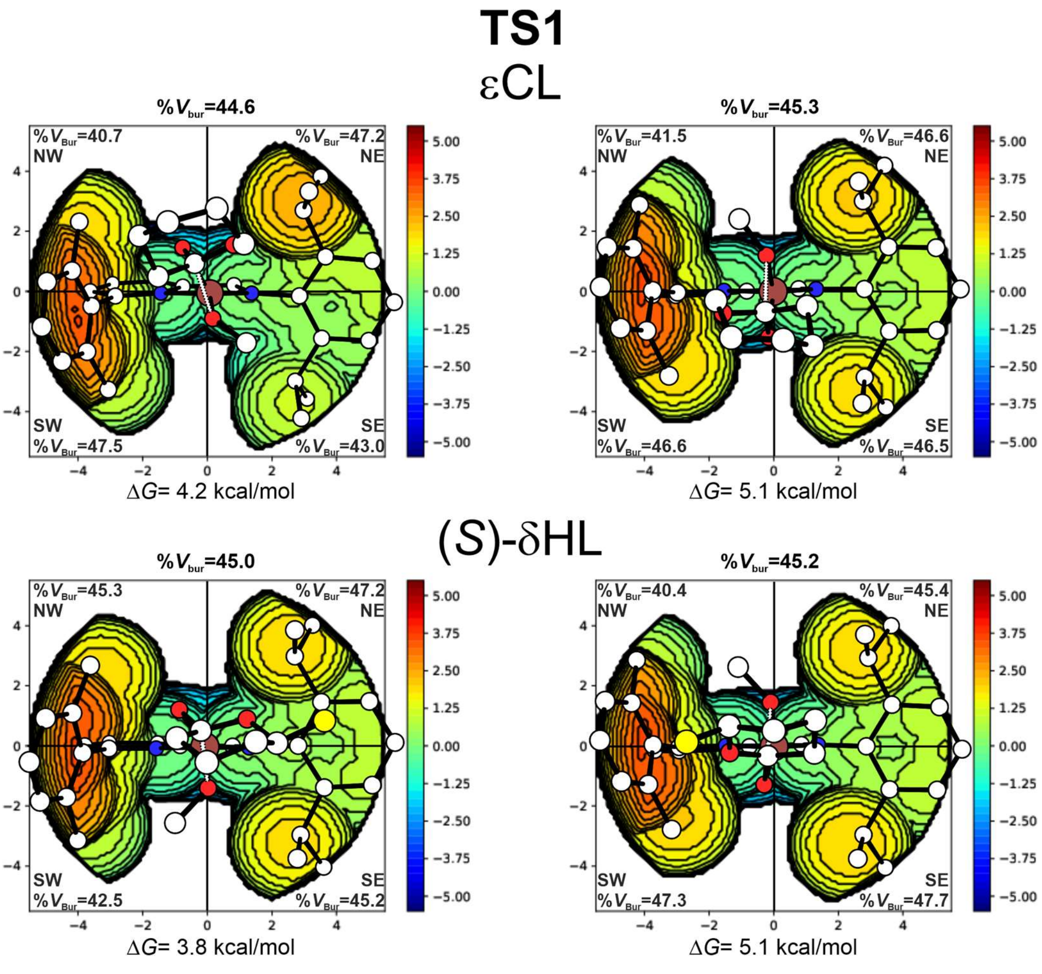

Figure S9. Steric maps of TS1 (nucleophilic attack) at both sites for εCL and δHL.

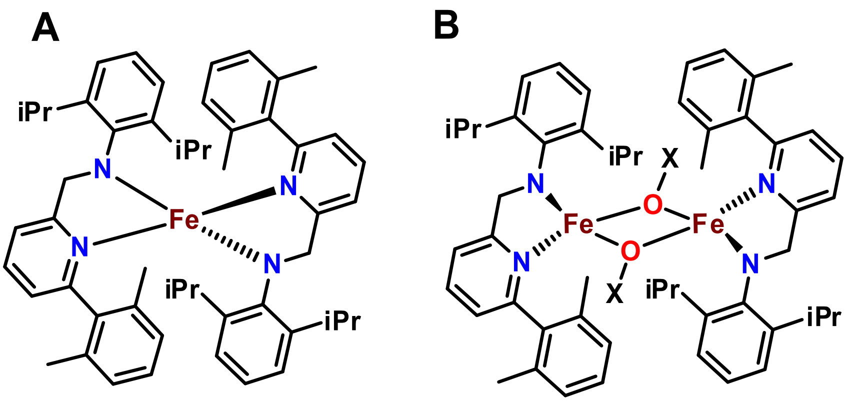

ΔG = -29.3 kcal/mol $\quad$ ΔG = -36.0 kcal/mol

Figure S10. Schematic representation of Homoleptic (**A**) and Dimeric (**B**) complexes obtained from the neutral precursor.

Below, the reaction schemes (RS1 and RS2) used for the energetic calculations for the formation of the homoleptic and dimeric species, respectively:

$$4\ LFeN(SiMe_3)_2 + MeOH \rightarrow 2\ L_2Fe + 4\ HN(SiMe_3)_2 + Fe_2(MeO)_4 \qquad \textbf{(RS1)}$$

$$2\ LFeN(SiMe_3)_2 + 2\ MeOH \rightarrow L_2FeFe_2(MeO)_4 + 2\ HN(SiMe_3)_2 \qquad \textbf{(RS2)}$$

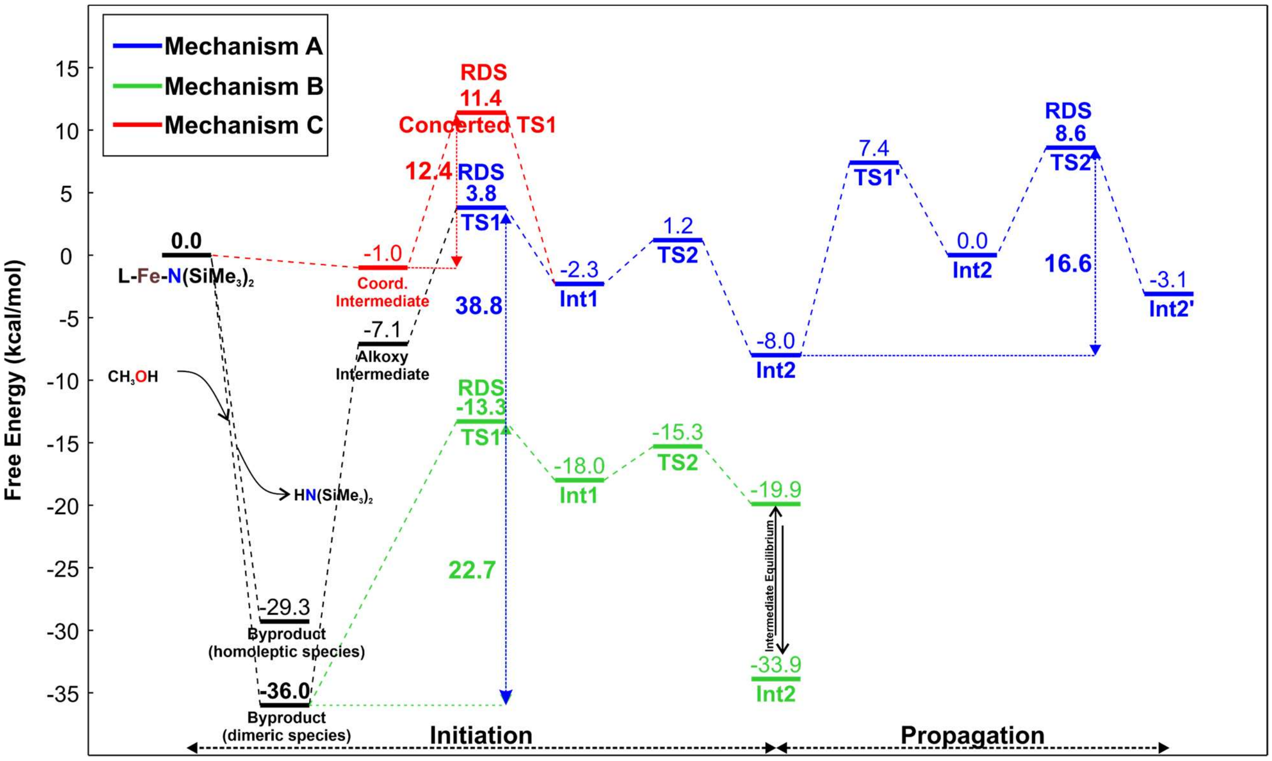

Figure S11. DFT computed Gibbs energetic profiles for ROP of δHL (initiation and propagation steps) following the Mechanism A (blue), B (green) and C (red). For discussion on the mechanisms see main text.

Table S6. Free energies in kcal/mol for δNL for critical point. All the values are reported with respect to the neutral precursor.

	**Concerted TS1**	**Int2**	**TS1'**	**TS2'**	**TS1 on Dimer**	**TS2 on Dimer**
ΔG (kcal/mol)	11.7	-8.5	8.1	10.1	-14.3	-16.4

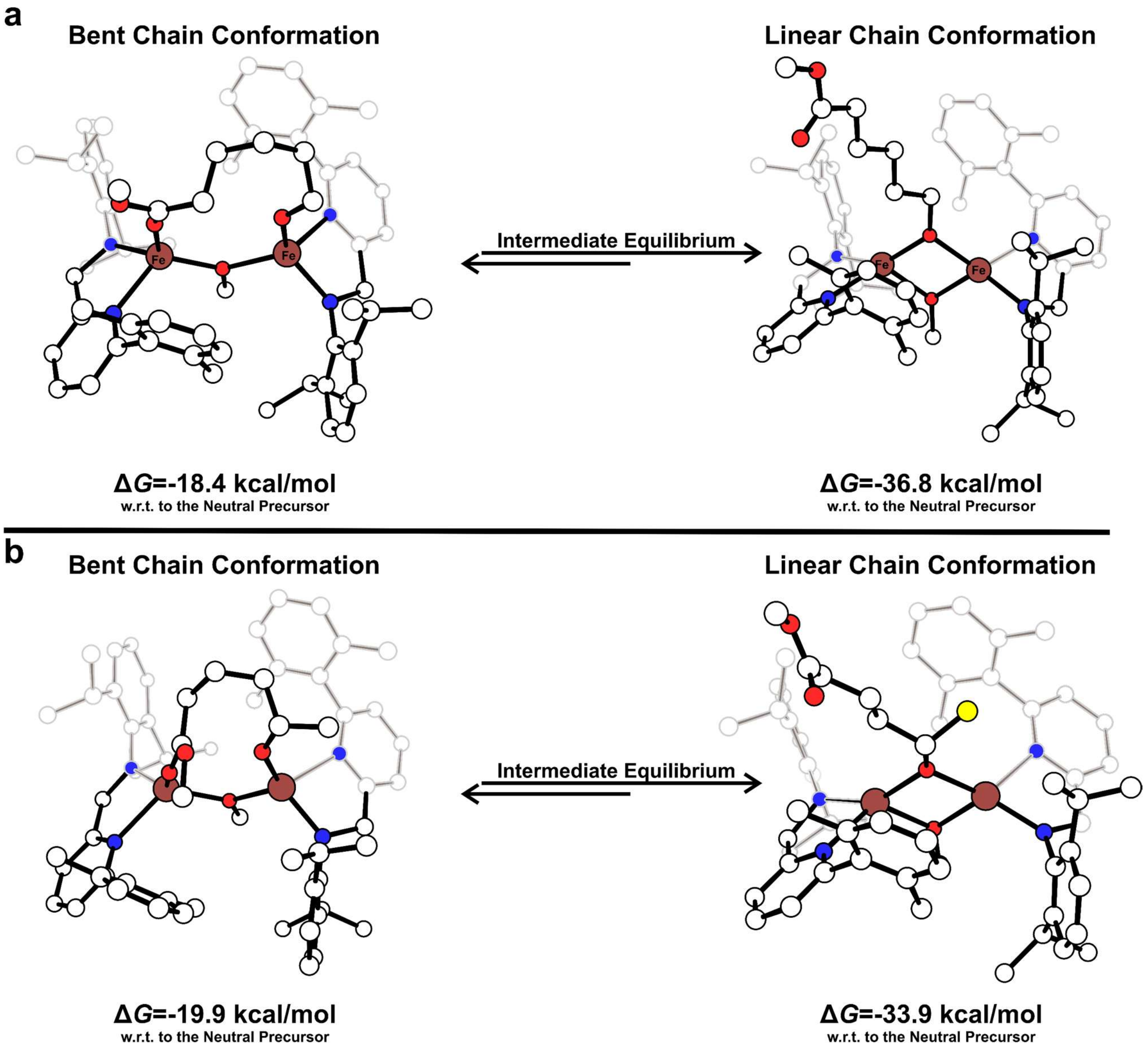

Figure S12. Equilibrium between growing chain Int2 structures on dimeric species for εCL (a) and δHL (b) in two different conformations, bent and linear. Gibbs energies are reported with respect to the neutral precursor (kcal/mol).

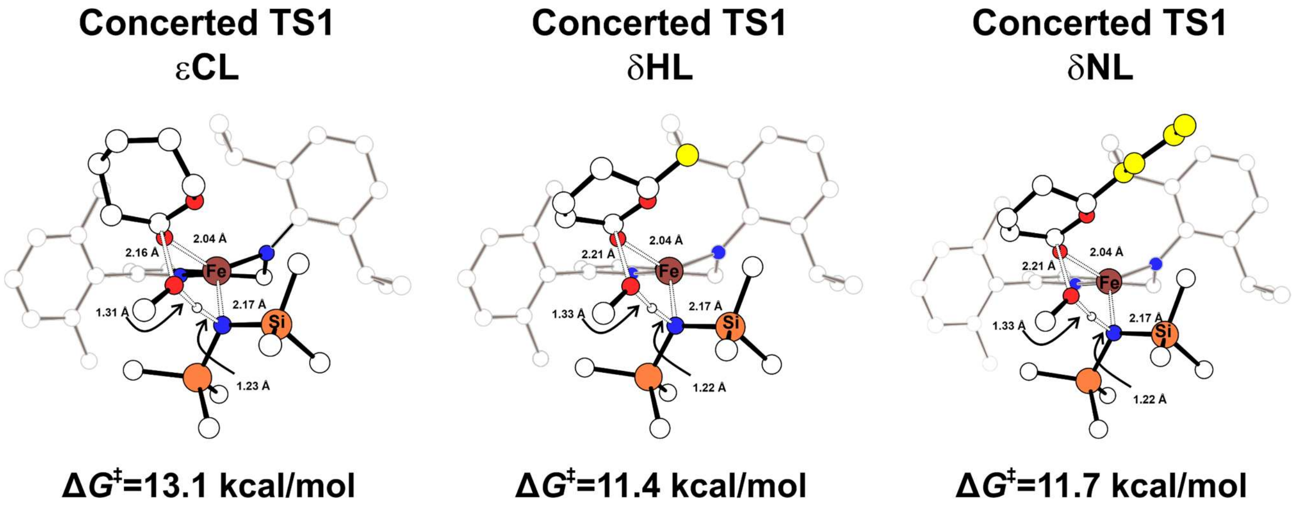

Figure S13. Concerted optimized TS structures (see Mechanism C in the main text) for εCL, δHL, and δNL respectively. The activation energies are reported in kcal/mol. All the distances are reported in Å.

Table S7. Experimental Gibbs energies (kcal/mol) derived from the experimental $k_{app.}$ ratio ($k_{app.(\delta HL)}/k_{app.(\varepsilon CL)}$ and $k_{app.(\delta NL)}/k_{app.(\varepsilon CL)}$), and DFT calculated energy activation barriers for monomeric and dimeric initiations and monomeric propagation using B3LYP approach.

	**ΔΔ*G*(δHL–εCL)**	**ΔΔ*G*(δNL–εCL)**
**Experimental**	1.4	2.6
**Monomeric Initiation**	-0.4	/
**Dimeric Initiation**	3.2	2.3
**Monomeric Propagation**	1.8	3.8

a) Calculated from the $k_{app.}$ ratio reported in Figure 2 by using the Arrhenius equation and compared to the $\Delta\Delta G^{\ddagger}_{calc}$. The reference used is the εCL and for the $\Delta\Delta G^{\ddagger}_{calc.}$ the activation energies of the initiation on the monomeric and dimeric specie and the propagation on the monomeric species have been considered.

Table S8. Free energies (ΔG in kcal/mol) for critical points of lactone MEPs, obtained with different functionals in single-point energy refinement calculations, reported with respect to the neutral monomeric precursor and compared to B3LYP-D3BJ.

	Initiation						Propagation		Methodology
	**TS1**	**TS2**	**Int2**	**Dimer precursor**	**TS1 on dimer**	**TS2 on dimer**	**TS1’**	**TS2’**	
**εCL**	4.2	2.5	-11.0	-36.0	-17.6	-16.6	3.8	3.5	B3LYP-D3BJ/PCM
	8.1	5.5	-9.5	-31.9	-13.2	-14.2	5.7	4.5	M06L-D3/PCM
	4.5	2.7	-8.2	-31.7	-12.6	-12.1	4.7	4.7	TPPSh-D3BJ/PCM
	5.1	2.7	-10.1	-34.9	-17.6	-16.6	4.5	3.6	ωB97XD/PCM
**δHL**	3.8	1.2	-8.0	-36.0	-13.3	-10.6	7.0	8.2	B3LYP-D3BJ/PCM
	6.2	5.0	-6.6	-31.9	-7.8	-12.1	9.6	10.9	M06L-D3/PCM
	4.0	1.5	-5.6	-31.7	-9.2	-11.1	7.3	9.5	TPPSh-D3BJ/PCM
	4.7	1.2	-6.9	-34.9	-13.6	-16.7	6.2	8.8	ωB97XD/PCM
**δNL**	/	/	-8.5	-36.0	-14.3	-16.4	8.1	10.1	B3LYP-D3BJ/PCM
	/	/	-7.6	-31.9	-9.2	-14.1	11.0	13.2	M06L-D3/PCM
	/	/	-6.3	-31.7	-10.3	-12.6	8.1	11.0	TPPSh-D3BJ/PCM
	/	/	-7.7	-34.9	-14.8	-18.4	6.9	9.6	ωB97XD/PCM

References

(1) Bandelli, D.; Weber, C.; Schubert, U. S. Strontium Isopropoxide: A Highly Active Catalyst for the Ring-Opening Polymerization of Lactide and Various Lactones. *Macromol. Rapid Commun.* **2019**, *40*, 1900306. https://doi.org/10.1002/marc.201900306.

(2) Duparc, V. H.; Shakaroun, R. M.; Slawinski, M.; Carpentier, J.-F.; Guillaume, S. M. Ring-Opening (Co)Polymerization of Six-Membered Substituted δ-Valerolactones with Alkali Metal Alkoxides. *Eur. Polym. J.* **2020**, *134*, 109858. https://doi.org/10.1016/j.eurpolymj.2020.109858.

(3) Bandelli, D.; Muljajew, I.; Scheuer, K.; Max, J. B.; Weber, C.; Schacher, F. H.; Jandt, K. D.; Schubert, U. S. Copolymerization of Caprolactone Isomers to Obtain Nanoparticles with Constant Hydrophobicity and Tunable Crystallinity. *Macromolecules* **2020**, *53*, 5208–5217. https://doi.org/10.1021/acs.macromol.0c00486.

(4) Zhao, J.; Hadjichristidis, N. Polymerization of 5-Alkyl δ-Lactones Catalyzed by Diphenyl Phosphate and Their Sequential Organocatalytic Polymerization with Monosubstituted Epoxides. *Polym Chem* **2015**, *6*, 2659–2668. https://doi.org/10.1039/C5PY00019J.

(5) Xu, C.; Wang, L.; Liu, Y.; Niu, H.; Shen, Y.; Li, Z. Rapid and Controlled Ring-Opening (Co)Polymerization of Bio-Sourced Alkyl-δ-Lactones To Produce Recyclable (Co)Polyesters and Their Application as Pressure-Sensitive Adhesives. *Macromolecules* **2023**, *56*, 6117–6125. https://doi.org/10.1021/acs.macromol.3c00920.

(6) Frisch, M. J.; Trucks, G. W.; Schlegel, H. B.; Scuseria, G. E.; Robb, M. A.; Cheeseman, J. R.; Scalmani, G.; Barone, V.; Petersson, G. A.; Nakatsuji, H.; Li, X.; Caricato, M.; Marenich, A. V.; Bloino, J.; Janesko, B. G.; Gomperts, R.; Mennucci, B.; Hratchian, H. P.; Ortiz, J. V.; Izmaylov, A. F.; Sonnenberg, J. L.; Williams; Ding, F.; Lipparini, F.; Egidi, F.; Goings, J.; Peng, B.; Petrone, A.; Henderson, T.; Ranasinghe, D.; Zakrzewski, V. G.; Gao, J.; Rega, N.; Zheng, G.; Liang, W.; Hada, M.; Ehara, M.; Toyota, K.; Fukuda, R.; Hasegawa, J.; Ishida, M.; Nakajima, T.; Honda, Y.; Kitao, O.; Nakai, H.; Vreven, T.; Throssell, K.; Montgomery Jr., J. A.; Peralta, J. E.; Ogliaro, F.; Bearpark, M. J.; Heyd, J. J.; Brothers, E. N.; Kudin, K. N.; Staroverov, V. N.; Keith, T. A.; Kobayashi, R.; Normand, J.; Raghavachari, K.; Rendell, A. P.; Burant, J. C.; Iyengar, S. S.; Tomasi, J.; Cossi, M.; Millam, J. M.; Klene, M.; Adamo, C.; Cammi, R.; Ochterski, J. W.; Martin, R. L.; Morokuma, K.; Farkas, O.; Foresman, J. B.; Fox, D. J. Gaussian 16 Rev. C.01, Wallingford, CT, 2016.

(7) Becke, A. D. Density-Functional Exchange-Energy Approximation with Correct Asymptotic Behavior. *Phys Rev A* **1988**, *38*, 3098–3100. https://doi.org/10.1103/PhysRevA.38.3098.

(8) Lee, C.; Yang, W.; Parr, R. G. Development of the Colle-Salvetti Correlation-Energy Formula into a Functional of the Electron Density. *Phys Rev B* **1988**, *37*, 785–789. https://doi.org/10.1103/PhysRevB.37.785.

(9) Hay, P. J.; Wadt, W. R. Ab Initio Effective Core Potentials for Molecular Calculations. Potentials for K to Au Including the Outermost Core Orbitals. *J. Chem. Phys.* **1985**, *82*, 299–310. https://doi.org/10.1063/1.448975.

(10) Schäfer, A.; Horn, H.; Ahlrichs, R. Fully Optimized Contracted Gaussian Basis Sets for Atoms Li to Kr. *J. Chem. Phys.* **1992**, *97*, 2571–2577. https://doi.org/10.1063/1.463096.

(11) Dunning, Thom. H.; Hay, P. J. Gaussian Basis Sets for Molecular Calculations. In *Methods of Electronic Structure Theory*; Schaefer, H. F., Ed.; Springer US: Boston, MA, 1977; pp 1–27. https://doi.org/10.1007/978-1-4757-0887-5_1.

(12) Cossi, M.; Barone, V.; Cammi, R.; Tomasi, J. Ab Initio Study of Solvated Molecules: A New Implementation of the Polarizable Continuum Model. *Chem. Phys. Lett.* **1996**, *255*, 327–335. https://doi.org/10.1016/0009-2614(96)00349-1.

(13) Grimme, S.; Ehrlich, S.; Goerigk, L. Effect of the Damping Function in Dispersion Corrected Density Functional Theory. *J. Comput. Chem.* **2011**, *32*, 1456–1465. https://doi.org/10.1002/jcc.21759.

(14) Wadt, W. R.; Hay, P. J. Ab Initio Effective Core Potentials for Molecular Calculations. Potentials for Main Group Elements Na to Bi. *J. Chem. Phys.* **1985**, *82*, 284–298. https://doi.org/10.1063/1.448800.
(15) McLean, A. D.; Chandler, G. S. Contracted Gaussian Basis Sets for Molecular Calculations. I. Second Row Atoms, Z=11-18. *J. Comput. Phys.* **1980**, *72*, 5639–5648. https://doi.org/10.1063/1.438980.
(16) Krishnan, R.; Binkley, J. S.; Seeger, R.; Pople, J. A. Self-consistent Molecular Orbital Methods. XX. A Basis Set for Correlated Wave Functions. *J. Chem. Phys.* **1980**, *72*, 650–654. https://doi.org/10.1063/1.438955.
(17) Zhao, Y.; Truhlar, D. G. The M06 Suite of Density Functionals for Main Group Thermochemistry, Thermochemical Kinetics, Noncovalent Interactions, Excited States, and Transition Elements: Two New Functionals and Systematic Testing of Four M06-Class Functionals and 12 Other Functionals. *Theor. Chem. Acc.* **2008**, *120*, 215–241. https://doi.org/10.1007/s00214-007-0310-x.
(18) Grimme, S. Accurate Description of van Der Waals Complexes by Density Functional Theory Including Empirical Corrections. *J. Comput. Chem.* **2004**, *25*, 1463–1473. https://doi.org/10.1002/jcc.20078.
(19) Adamo, C.; Barone, V. Toward Reliable Density Functional Methods without Adjustable Parameters: The PBE0 Model. *J. Chem. Phys.* **1999**, *110*, 6158–6170. https://doi.org/10.1063/1.478522.
(20) Ernzerhof, M.; Scuseria, G. E. Assessment of the Perdew–Burke–Ernzerhof Exchange-Correlation Functional. *J. Chem. Phys.* **1999**, *110*, 5029–5036. https://doi.org/10.1063/1.478401.
(21) Tao, J.; Perdew, J. P.; Staroverov, V. N.; Scuseria, G. E. Climbing the Density Functional Ladder: Nonempirical Meta–Generalized Gradient Approximation Designed for Molecules and Solids. *Phys Rev Lett* **2003**, *91*, 146401. https://doi.org/10.1103/PhysRevLett.91.146401.
(22) Chai, J.-D.; Head-Gordon, M. Long-Range Corrected Hybrid Density Functionals with Damped Atom–Atom Dispersion Corrections. *Phys Chem Chem Phys* **2008**, *10*, 6615–6620. https://doi.org/10.1039/B810189B.
(23) Falivene, L.; Cao, Z.; Petta, A.; Serra, L.; Poater, A.; Oliva, R.; Scarano, V.; Cavallo, L. Towards the Online Computer-Aided Design of Catalytic Pockets. *Nat. Chem.* **2019**, *11*, 872–879. https://doi.org/10.1038/s41557-019-0319-5.
(24) Boys, S. F.; Bernardi, F. The Calculation of Small Molecular Interactions by the Differences of Separate Total Energies. Some Procedures with Reduced Errors. *Mol. Phys.* **1970**, *19*, 553–566. https://doi.org/10.1080/00268977000101561.
